# Genetic Control of Myelin Plasticity after Chronic Psychosocial Stress

**DOI:** 10.1523/ENEURO.0166-18.2018

**Published:** 2018-07-11

**Authors:** Mikaela A. Laine, Kalevi Trontti, Zuzanna Misiewicz, Ewa Sokolowska, Natalia Kulesskaya, Aino Heikkinen, Suvi Saarnio, Ingrid Balcells, Pierre Ameslon, Dario Greco, Pirkko Mattila, Pekka Ellonen, Lars Paulin, Petri Auvinen, Eija Jokitalo, Iiris Hovatta

**Affiliations:** 1 Molecular and Integrative Biosciences Research Program, University of Helsinki, Helsinki FI-00014, Finland; 2 Institute of Biotechnology, University of Helsinki, Helsinki FI-00014, Finland; 3 Finnish Institute of Molecular Medicine, University of Helsinki, Helsinki FI-00014, Finland

**Keywords:** anxiety, chronic social defeat stress, inbred mouse strain, myelin, RNA-sequencing, transmission electron microscopy

## Abstract

Anxiety disorders often manifest in genetically susceptible individuals after psychosocial stress, but the mechanisms underlying these gene-environment interactions are largely unknown. We used the chronic social defeat stress (CSDS) mouse model to study resilience and susceptibility to chronic psychosocial stress. We identified a strong genetic background effect in CSDS-induced social avoidance (SA) using four inbred mouse strains: 69% of C57BL/6NCrl (B6), 23% of BALB/cAnNCrl, 19% of 129S2/SvPasCrl, and 5% of DBA/2NCrl (D2) mice were stress resilient. Furthermore, different inbred mouse strains responded differently to stress, suggesting they use distinct coping strategies. To identify biological pathways affected by CSDS, we used RNA-sequencing (RNA-seq) of three brain regions of two strains, B6 and D2: medial prefrontal cortex (mPFC), ventral hippocampus (vHPC), and bed nucleus of the stria terminalis (BNST). We discovered overrepresentation of oligodendrocyte (OLG)-related genes in the differentially expressed gene population. Because OLGs myelinate axons, we measured myelin thickness and found significant region and strain-specific differences. For example, in resilient D2 mice, mPFC axons had thinner myelin than controls, whereas susceptible B6 mice had thinner myelin than controls in the vHPC. Neither myelin-related gene expression in several other regions nor corpus callosum thickness differed between stressed and control animals. Our unbiased gene expression experiment suggests that myelin plasticity is a substantial response to chronic psychosocial stress, varies across brain regions, and is genetically controlled. Identification of genetic regulators of the myelin response will provide mechanistic insight into the molecular basis of stress-related diseases, such as anxiety disorders, a critical step in developing targeted therapy.

## Visual Abstract

**Figure:**
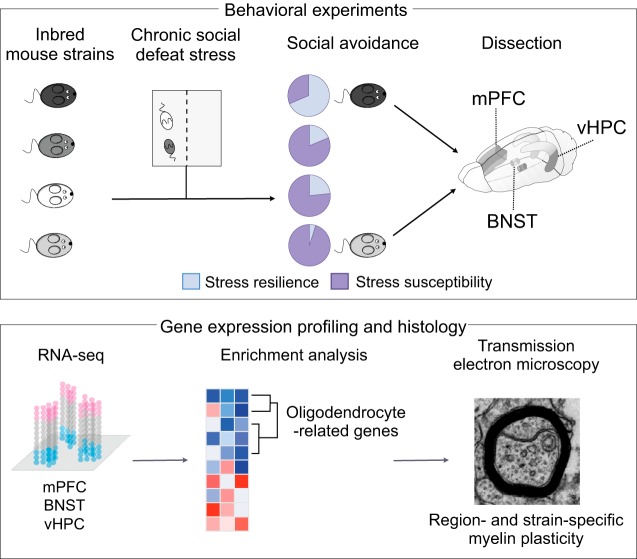

## Significance Statement

Chronic psychosocial stress is a well-established risk factor for anxiety disorders, but the development of targets for therapeutic intervention is limited by ignorance of the underlying molecular and cellular mechanisms. We used inbred genetically defined mice to identify neurobiological pathways that underlie stress-induced social avoidance (SA), a type of anxiety. We found genetically controlled differences in myelin-related gene expression in stress-exposed mice, with concurrent differences in myelin thickness, suggesting that myelin plasticity is a major stress response of the brain. The adaptive response to stress may increase or decrease myelin thickness, depending on the demands of the specific circuit. Our findings provide a foundation for the identification of specific genetic regulators of chronic stress-induced myelin plasticity.

## Introduction

Anxiety disorders, including panic disorder, social anxiety disorder, specific phobias, and generalized anxiety disorder, are the most common mental disorders with a prevalence of 14% (Wittchen et al., 2011). Human genetic studies of anxiety disorders have confirmed a modest heritability and considerable environmental component (Hettema et al., 2001). However, identification of replicated risk variants is challenging due to genetic heterogeneity, environmental factors that cannot be well-controlled in human settings, and poor availability of large patient cohorts with accurate phenotypes (Hovatta and Barlow, 2008; Smoller, 2016). Consequently, many investigators have resorted to animal models, which allow controlled experiments on both genetic and environmental risk factors, to reveal the biological mechanisms underlying these diseases.

Chronic psychosocial stress is a well-established risk factor for anxiety disorders (Kemeny and Schedlowski, 2007; Moffitt et al., 2007; Donner et al., 2012). It can be modeled in mice by the chronic social defeat stress (CSDS) paradigm, that has etiological, predictive, and face validity for affective and anxiety disorders (Hammels et al., 2015). It leads to social avoidance (SA) and long-term plastic changes in the brain (Avgustinovich et al., 2005; Krishnan et al., 2007). However, only a portion of mice exhibit SA after CSDS, and thus the defeated animals can be divided into stress-susceptible and resilient. Comparison of these groups allows investigation of the mechanistic basis of stress-induced SA and anxiety-like behavior and resilience to it. Understanding the underlying risk factors and promotion of the resilience factors in susceptible individuals should facilitate development of secondary prevention methods of anxiety disorders after traumatic events, and selective pharmacological treatment of anxiety (Howlett and Stein, 2016).

Most insight into the molecular mechanisms of CSDS comes from the C57BL/6 mouse strain, in which specific genetic, epigenetic, and neurophysiological mechanisms underlie stress susceptibility and resilience (Han and Nestler, 2017). Because genetic background strongly modulates mouse behavior (Threadgill et al., 1995; Hovatta et al., 2005; Sittig et al., 2016), hypotheses of any mechanisms should be tested across different mouse strains. Such studies are also critical for translating the results to genetically heterogeneous humans. CSDS has different behavioral and physiologic consequences in C57BL/6J and BALB/c strains (Razzoli et al., 2011a,b; Savignac et al., 2011), reflecting underlying genetic, and by extension, gene expression differences between the strains. Therefore, comprehensive unbiased transcriptomic approaches applied to mouse strains with different responses to CSDS should reveal gene-environment interactions that explain why some individuals are more susceptible to stress-induced maladaptive behaviors than others.

To examine how genetic background affects the behavioral response to chronic stress, we performed behavioral testing in four inbred mouse strains after CSDS. We selected C57BL/6NCrl (B6) and BALB/cAnNCrl (BALB) strains, which differ in behavioral and metabolic responses to CSDS (Razzoli et al., 2011a,b; Savignac et al., 2011), and DBA/2NCrl (D2) and 129S2/SvPasCrl (129) as they are sensitive to stress-induced anxiety- and depression-like behavior in general (Ducottet and Belzung, 2005; Millstein and Holmes, 2007). To identify the major underlying biological pathways, we performed unbiased transcriptomic analysis in the B6 and D2 strains that showed the largest differences in susceptibility to stress. We studied three brain regions, the medial prefrontal cortex (mPFC), ventral hippocampus (vHPC), and bed nucleus of the stria terminalis (BNST), which critically regulate anxiety and the stress response (Garakani et al., 2009; Calhoon and Tye, 2015; Tovote et al., 2015), and are activated by CSDS (Vialou et al., 2014; Laine et al., 2017). In the follow-up gene expression and histologic analyses, we found that myelination was significantly altered after stress in a strain and brain region-dependent manner. Our results illustrate that genetic background has a large effect on both the behavioral and brain transcriptomic response to chronic psychosocial stress. Moreover, we demonstrate that the pattern of stress-induced myelination changes is dependent on the genetic background and varies across brain regions.

## Materials and Methods

### Animals

We ordered five-week-old male mice from four inbred strains [DBA/2NCrl (D2), 129S2/SvPasCrl (129), BALB/cAnNCrl (BALB), and C57BL/6NCrl (B6); Charles River Laboratories] for all CSDS experiments and let them acclimatize for 10 d before CSDS, housed in groups in a temperature (22 ± 2°C) and humidity (50 ± 15%) controlled facility on a 12/12 h light/dark cycle (lights on 6 A.M. to 6 P.M.). From the end of CSDS to the time of dissection, all mice were single-housed. As aggressors for CSDS, we used male Clr-CD1 mice (CD1, Charles River Laboratories), aged 13–26 weeks. All mice had ad libitum access to food and water throughout the experiment, except for the durations of behavioral tests. Aspen chip bedding (Tapvei Oy) in the cages was changed weekly (except for the duration of the CSDS) and standard environmental enrichment [aspen strips as nesting material (Tapvei Oy) and an aspen brick (Tapvei Oy)] was provided throughout the experiment. Animal procedures were approved by the Regional State Administration Agency for Southern Finland (ESAVI-3801-041003-2011 and ESAVI/2766/04.10.07/2014) and conducted in accordance to directive 2010/63/EU of the European Parliament and of the Council, and the Finnish Act on the Protection of Animals Used for Science or Educational Purposes (497/2013).

### Behavioral experiments

#### CSDS

We conducted CSDS as previously described (Golden et al., 2011; Laine et al., 2017). Briefly, aggressor CD1 mice were first checked for appropriate aggression levels during a 3-d screening before all social defeat experiments. The selected aggressors had to meet the following criteria: attack in at least two consecutive sessions, had latency to attack of <90 s and do not attack within 1–5 s in any of the sessions. For CSDS, each defeated mouse was placed into the cage of an aggressor mouse for max. 10 min. The mouse was then transferred to another compartment of the cage, separated from the aggressor by a perforated Plexiglas wall, for 24 h. This procedure was repeated for 10 d, and each day the experimental mouse encountered a novel aggressor. Physical contact time was shortened in the case of severe physical aggression. Control mice were housed in similar cages but with another control mouse as a cage-mate, and without physical contact, switching cage-mates daily. The day after the last defeat session, all mice were separated into single-housed cages and maintained like this until dissection. The order of testing for all consecutive behavioural tests was randomized.

#### SA test

Twenty-four hours after the last social defeat session, at the start of the dark phase of the light cycle, we tested both defeated and control mice in the SA test. All animals were brought to the experiment room at least 30 min before the start of the test, and animals performing the test were separated from experimenters and the remaining animals by a screen. For the first trial (no-target), the mouse was placed in the center of an open arena (42 × 42 cm) with an empty perforated Plexiglas cylinder located next to one of the walls. The movements of the mouse were tracked using a camera and EthoVision XT10 software (Noldus Information Technology) for 150 s, after which the mouse was placed back into the home cage. The arena was cleaned and the Plexiglas cylinder was replaced with another one containing an unfamiliar CD1 social target mouse. The test mouse was then placed back in the middle of the arena and tracked for 150 s (target trial). The amount of time the mice spent in the interaction zone (IZ), defined as a semicircle (370 cm^2^) around the perforated Plexiglas cylinder, was measured and a social interaction (SI) ratio calculated by dividing the IZ time of the social target trial with the IZ time of the no-target trial, multiplied by 100. Thus, a low SI ratio indicates high SA.

To account for strain differences in baseline social behavior, we assessed the response of defeated mice in relation to same-strain controls, following the statistical approach previously implemented by Nasca et al. (2015). We calculated mean SI ratios of control mice from each strain based on several cohorts (*n*: 129 = 8, BALB = 40, B6 = 126, D2 = 114). We used log-transformation to normalize the distribution, removed outliers (>3 IQRs from the median), and divided the defeated mice to stress-resilient (resembling controls) and susceptible (showing SA) based on SI ratios, with the border determined as the controls’ mean score minus 1 SD. SI ratio border values for each strain were: 129 = 62.68, BALB = 81.76, B6 = 76.49, D2 = 105.99.

#### Body weight

Body weight was recorded at the beginning of CSDS (day 1) and on every second day throughout the CSDS (days 2, 4, 6, 8, and 10). The amount of body weight gain was calculated as the difference between the first and the last measurement.

#### Open field (OF) test

We assessed spontaneous locomotor activity and anxiety-like behavior with an automatic MedAssociates system at the start of the light phase of the light cycle. Individual mouse cages were brought to the experimental room in two groups. After 30 min adaptation to the experimental room (lit at 100 lux), the mouse was released to the corner of the experimental chamber (27 × 27 × 20 cm, transparent walls and white floor virtually divided into a 19 × 19 squares grid) and allowed to explore freely for 5 min.

#### Elevated zero maze (EZM)

EZM was performed at the start of the light phase of the light cycle. All animals were brought to the experiment room at least 30 min before the test, and animals performing the test were separated from experimenters and the remaining animals by a screen. The apparatus consisted of plastic annular runway (diameter = 50 cm, width = 5 cm) elevated 40 cm above the floor. The runway was divided into four sectors: two open sectors opposing each other and two opposing closed sectors protected by inner and outer non-transparent walls (height = 15 cm). After 30 min of adaptation to the experimental room (dimly lit at 15–20 lux), the mouse was placed in the middle of one of the closed sectors and allowed to explore the maze freely for 5 min. The mouse was video-tracked with EthoVision XT10.

#### Forced swim test (FST)

All animals were brought to the experiment room at least 30 min before the test, and animals performing the test were separated from experimenters and the remaining animals by a screen. After adaptation to the experimental room (150 lux), the mouse was placed in a glass cylinder (diameter = 18 cm, height = 25 cm) filled with water (room temperature) to the height of 15 cm. The immobility time (passive floating) was detected with EthoVision XT10 system for 6 min with 2-min time bins. Data from the last 4 min were used.

### Gene expression profiling

#### Dissections

We dissected the mPFC (B6: Con *n* = 6, Res *n* = 6, Sus *n* = 6; D2: Con *n* = 6, Sus *n* = 8), BNST (B6: Con *n* = 5, Res *n* = 5, Sus *n* = 5; D2: Con *n* = 5, Res *n* = 3, Sus *n* = 5), and vHPC (B6: Con *n* = 6, Res *n* = 8, Sus *n* = 3; D2: Con *n* = 6, Sus *n* = 5) 6–8 d after the last CSDS (mice aged eight weeks). Mice were killed by cervical dislocation between 8 and 11 A.M. to avoid circadian differences in gene expression, and the order was counterbalanced across groups (resilient, susceptible, and control mice). Dissections were performed on a sterile chilled Petri dish within 7 min, and tissue was flash frozen in liquid N_2_.

#### RNA-sequencing (RNA-seq)

Total RNA was extracted with TriReagent (Molecular Research Center Inc.) and RNA quality was assessed with a 2100 Bioanalyzer (Agilent Technologies) using Agilent RNA 600 Nano Chip kit (Agilent Technologies). rRNA was depleted with Ribo-Zero Gold rRNA Removal kit (Illumina Inc; mPFC and vHPC) or custom Insert Dependent Adaptor Cleavage (InDA-C) primers (BNST). RNA was fragmented using the S2 ultrasonicator (Covaris Inc.) and sequencing libraries were prepared with Nextera (Illumina; vHPC), ScriptSeq v2 (Epicentre; mPFC), or Ovation Universal RNA-Seq System (NuGEN; BNST) RNA-seq library preparation kits. Libraries were size-selected with Pippin Prep (Sage Science) and sequencing was conducted on HighSeq 2000 (vHPC, paired-end 91 bp, Illumina) or NextSeq 500 platforms (mPFC and BNST, single-end 96 bp; Illumina).

The RNA-seq reads were trimmed for adapters with Cutadapt v1.8.3 (vHPC) and FastX toolkit (mPFC, BNST) and PCR duplicates were removed with PRINSEQ v0.20.4. Reads were aligned using STARv 2.5.0c (Dobin et al., 2013) with default settings to mouse genome GRCm38, and annotated to gene exons with HTSeq v0.6.1 (Anders et al., 2015) using GTF release 86 (update 2016-10).

#### Differential expression (DE) analysis

DE analysis was conducted using limma eBayes (Ritchie et al., 2015; Phipson et al., 2016) comparing resilient and susceptible mice to same-strain controls within brain regions. RNA-seq data were filtered to remove low-abundance genes, keeping genes with at least 1 count per million (CPM) in at least six samples (Anders et al., 2015). Subsequently, the data were normalized with voom (Law et al., 2014), and adjusted for sequencing (vHPC) and library preparation batches (vHPC, mPFC, and BNST) with ComBat (Johnson et al., 2007). To identify top DE genes, we calculated -log10(*p*)*logFC for each gene and ranked them accordingly (Xiao et al., 2014).

Gene expression data are available in Gene Expression Omnibus (GEO; GSE109315).

#### Rank-rank hypergeometric overlap (RRHO) test (Plaisier et al., 2010)

RRHO infers pair-wise similarity between two DE result lists, where genes are ranked by DE (-log10(*p*)*logFC) between resilient and control, or between susceptible and control mice, by calculating significance of overlapping genes at different rank bins (Plaisier et al., 2010). We used step size of 100 genes to bin the genes and applied the same -log(*p*) scale to comparisons within brain regions. Significant overlap of genes in rank groups containing up- and downregulated genes in both lists (lower left corner and upper right corner of square matrix, respectively, see Fig. 2*C*,*D*) shows a shared transcriptome-wide gene expression pattern in response to CSDS.

#### Gene set enrichment analysis (GSEA)

We conducted GSEA using the GSEA Preranked module implemented in GSEA Desktop v3.0 (Mootha et al., 2003; Subramanian et al., 2005) and the curated gene sets (C2) of the Molecular Signature Database (MSigDB) v6.0 (http://www.broad.mit.edu/gsea/). The pre-ranked GSEA was performed with 1000 permutations. The top five gene sets with the highest positive and negative normalized enrichment scores (NESs; *p_FDR_* < 0.05) within each comparison were selected for further analysis. From the selected top up- and downregulated gene sets, all present in at least two comparisons and two brain regions were visualized using Circos software (Krzywinski et al., 2009). The overlap between the top enriched gene sets, presented on the Circos plot, was further investigated with the hypergeometric test implemented in the MSigDB v6.0.

#### Gene ontology (GO) term enrichment

We analyzed GO term enrichment separately for top 300 upregulated and 300 downregulated differentially expressed genes from each comparison with topGO (Alexa et al., 2006), using the weight01 model to account for GO term dependencies. In RNA-seq, long transcripts yield more read counts and are more easily passed through low-abundance filtering than short genes with the same expression level, and genes with high number of counts have greater statistical power being detected as DE than genes with low number of counts. To minimize these selection biases (Young et al., 2010), we matched the background genes, i.e., the gene universe used to compare the top DE genes with, with the top DE genes using R package genefilter (Gentleman et al., 2017), resulting in 6882 (±290 SEM) background genes.

#### Visualizing oligodendrocyte (OLG) progenitor cell (OPC) and OLG marker genes

We manually curated a list of OPC and OLG-specific marker genes, based on prior publications (Zhang et al., 2014; Marques et al., 2016). Figures were constructed based on previously published scripts (Haarman et al., 2014).

#### q-RT-PCR

q-RT-PCR was applied to validate five myelin-related genes from RNA-seq. We used published primers to amplify *Mobp* and *Plp1* (Liu et al., 2012) and designed primer pairs (5’-3’ forward, reverse) using NCBI primer designing tool (https://www.ncbi.nlm.nih.gov/tools/primer-blast/) for *Opalin* (ACTGCCATCGAATACGACATC, CCTCTACGGGCTCATCATCG), *Ermn* (AACCAGGCAGGAGACAACTG, GATGGCCTGGTGAACAACGA), and *Mbp* (ACACACGAGAACTACCCATTATGG, AGAAATGGACTACTGGGTTTTCATCT). RNA samples of mPFC and BNST were the same as used in RNA-seq, and for vHPC samples overlapped by 37.5% (12/32 samples); 250 ng of DNase I (Thermo Scientific)-treated total RNA was converted to cDNA with iScript select cDNA synthesis kit (Bio-Rad Laboratories) and amplified with 250 nM primers in CFX384 Real-Time PCR cycler using IQ SYBR Green supermix (Bio-Rad Laboratories). Expression levels were normalized to *Ppib* (GGAGATGGCACAGGAGGAAA, CCCGTAGTGCTTCAGCTTGAA). Each reaction was run in triplicate and relative expression level was calculated using a standard curve (7.15, 10.0, 5.0, 2.0, 1.0, 0.5, and 0.25 ng of cDNA) present on each assay plate with CFX Manager (Bio-Rad Laboratories). Statistical analysis (Pearson’s *r*) was conducted with GraphPad Prism v7.02 (GraphPad Software Inc.).

### Immunohistochemistry

Mice were anaesthetized 6–8 d after CSDS with a lethal dose of pentobarbital (Mebunat Vet 60 mg/ml, Orion Pharma) and transcardially perfused with 4% paraformaldehyde (PFA) in PBS. After postfixation in 4% PFA overnight (+4°C), the brains were cut into 20-µm coronal sections using a Leica VT-1200S vibratome (Leica Biosystems) and stored at –20°C free-floating in cryoprotectant. Sections were washed 3× in PBS and mounted on Superfrost Ultra Plus (ThermoFisher Scientific) slides. We performed antigen retrieval by submerging the slides in 0.01 M citrate buffer, heated to a boil for 20 min. Slides were blocked in 2.5% BSA in 0.5% PBST + 7.5% normal goat serum, followed by primary antibody incubation with mouse anti-CNPase (1:250, Merck Life Science, #MAB326R) overnight at +4°C. Slides were washed in PBS before goat anti-mouse Alexa Fluor 488 secondary antibody incubation (1:400, ThermoFisher Scientific, #A-11029) for 2 h at room temerture. After washing in PBS, we coverslipped the slides with Vectashield + DAPI mounting medium (Vector Laboratories, #H-1200). We acquired images with ZEISS Apotome.2 system (Zeiss) and analyzed them with ImageJ software (National Institutes of Health). We measured corpus callosum thickness on both sides of the midline from each section (two to six sections per animal, distance from bregma between 0.22 and -0.10) using ImageJ, and calculated their mean.

### Transmission electron microscopy (TEM)

Mice were anaesthetized 6–8 d after CSDS with a lethal dose of pentobarbital (Mebunat Vet). We transcardially perfused the mice with PBS followed by fixation with 100 ml 2% glutaraldehyde (GA)/2% PFA in 0.1 M sodium cacodylate (NaCac) buffer (GA, PFA, and NaCac: Sigma Aldrich), heated to +37°C. The brains were postfixed in the same fixative for 2–4 h and immersed in 0.1 M NaCac buffer for 2–24 h, both at +4°C. We cut them into 200-μm sagittal slices with a Leica VT-1200S vibratome (Leica Biosystems) in 0.1 M phosphate buffer. Regions of the mPFC, BNST, and vHPC were cut manually by using anatomic landmarks (Extended Data Fig. 5-1).

We postfixed the sections in osmium tetroxide [1%, +1.5% K4[Fe(CN)6] in 0.1 M NaCac] for 2 h at +4°C, stained en bloc with uranyl acetate for 1 h at +4°C, dehydrated with EtOH and acetone, and embedded them into hard Epon; 60- to 70-nm sections were cut with an ultramicrotome and placed on copper grids for microscopy and stained with lead citrate. We imaged the ultrathin sections using a Jem-1400 transmission electron microscope (Jeol) and randomly selected and imaged myelinated axons at 5000× magnification.

We measured myelin thickness, axon diameter, and g ratio using ImageJ. We calculated the diameter by measuring the area of the whole fiber and the area of the axon (inside the compacted myelin sheath). The diameters of geometric circles with the same areas were calculated for both parameters. We calculated the g ratio by dividing the diameter of the axon with the diameter of the whole fiber. Myelin thickness was measured at three fully compacted positions and their average calculated.

### Statistical analysis

Statistical analyses were conducted with SPSS Statistics 24 (IBM) or GraphPad Prism 7.02 (GraphPad Software Inc.). Planned *post hoc* comparisons (control vs resilient, control vs susceptible, and resilient vs susceptible) were conducted by Fisher’s LSD. For these tests we report nominal *p* values, evaluated for significance against an α-level adjusted for multiple corrections with test-wise Bonferroni correction (Table 1). Only *p* values which survive this correction are shown.

**Table 1.** Statistical table

Figure	Data structure	Statistical test	Statistical significance (α)
1*B*	Categorical data	χ^2^	0.05
1*C*	Normal distribution	Mixed ANOVA	0.0167
1*D*	Normal distribution	Mixed ANOVA	0.0167
1*E*	Normal distribution	One-way ANOVA	0.0167
1*F*	Normal distribution	One-way ANOVA (129, BALB and B6 strains), independent *t* test (D2 strain)	0.0167
1*G*	Normal distribution	One-way ANOVA	0.0167
1*H*	Normal distribution	One-way ANOVA	0.0167
1*I*	Normal distribution	One-way ANOVA (group comparisons), mixed ANOVA (within-group comparison of weight before and after CSDS)	0.0167
4*A–F*	Normal distribution	Mixed ANOVA	0.003 (B6) 0.01 (D2)
4*H*,*I*	Normal distribution	One-way ANOVA	0.0167
5*A–L*	Normal distribution	Generalized estimating equations (GEEs)	0.0167

Outline of statistical tests and significance levels applied for each experiment.

Group differences in TEM data were assessed using generalized estimating equations (GEEs) to control for within-subject dependencies of individual axons measured from the same animal (ranges for each region: mPFC = 93–104, BNST = 31–69, and vHPC = 54–62 axons per animal). We selected this approach due to the low number of animals per group, which could not be reliably analyzed by ANOVA. GEE has been proposed as a suitable approach for analyzing data with non-independent features (Hanley et al., 2003), such as axons measured from the same individual. Pair-wise contrasts were computed for comparing groups with Fisher’s LSD and significance determined against the Bonferroni corrected α-level as above.

Data analysis of RNA-seq data were performed as described above. Multiple testing correction was done by the Benjamini–Hochberg method (Benjamini and Hochberg, 1995).

## Results

### Genetic background influences behavioral response to CSDS

**Figure 1.**
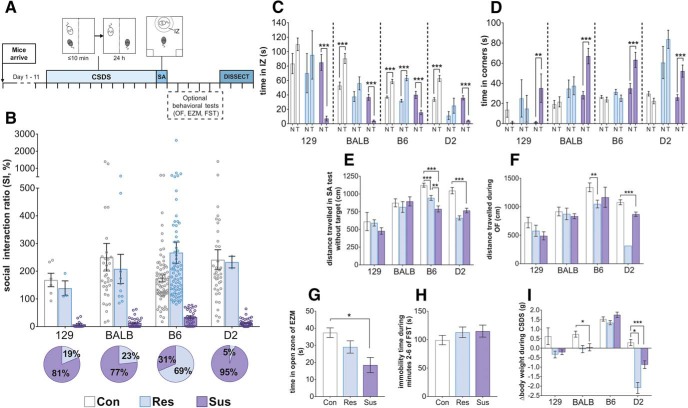
Strong genetic background effect on the behavioral response to CSDS. ***A***, Timeline of experiments. Each perpendicular line represents 1 d. ***B***, SI ratios of the four strains in the SA test. Pie charts represent the proportion of resilient and susceptible mice in each mouse strain. 129, BALB, and D2 strains were highly susceptible to CSDS, while B6 strain was the most resilient to CSDS. Time spent in the IZ (***C***) and the corner zones (***D***) of the SA test during sessions with no social target (N) and a CD1 mouse as a social target (T). Susceptible mice of all strains spent less time in the IZ when the target was present compared to when it was not. ***E***, Distance traveled during the no-target trial of the SA test. In the B6 strain, both susceptible and resilient mice had lower locomotor activity, while in the D2 strain only susceptible mice moved significantly less than controls; *n* (***B–E***, ***I***) = 129: Con = 7, Res = 3, Sus = 13; C: Con = 34, Res = 10, Sus = 33; B6: Con = 72, Res = 70, Sus = 32; D2: Con = 39, Res = 2, Sus = 40. ***F***, Distance traveled during the 5-min OF test. B6 resilient and D2 susceptible mice traveled significantly shorter distances than their same-strain control mice; *n* = 129: Con = 7, Res = 3, Sus = 13; C: Con = 18, Res = 6, Sus = 19; B6: Con = 20, Res = 19, Sus = 4; D2: Con = 19, Res = 1 (not analyzed), Sus = 22. ***G***, Time B6 mice spent in the open area of the EZM. Susceptible mice spent significantly less time in the open zones compared to controls; *n*: Con = 20, Res = 29, Sus = 11. ***H***, Immobility time of B6 mice during minutes 2–6 of the FST did not differ between groups; *n* = Con = 28, Res = 32, Sus = 21. ***I***, Difference in body weight before and after CSDS. B6 defeated mice gained weight during CSDS similarly to their same-strain controls, BALB susceptible mice gained significantly less weight than controls, and both resilient and susceptible D2 mice lost weight. All figures depict mean ± 1 SEM; *p < 0.05, **p < 0.01, ***p < 0.001, see Extended Data Figure 1-1 for exact *p* values. D2: DBA/2NCrl strain; BALB: Balb/cAnNCrl; 129: 129S2/SvPasCrl; B6: C57BL/6NCrl; Con: control; Res: resilient; Sus: susceptible; mPFC: medial prefrontal cortex; BNST: bed nucleus of the stria terminalis; vHPC: ventral hippocampus.

To determine whether genetic background affects the behavioral response to psychosocial stress, we conducted 10-d CSDS in mice from four inbred strains, D2, 129, BALB, and B6 (Fig. 1*A*). Twenty-four hours after the last defeat session, we conducted the SA test to assess their SA phenotype. To account for the strain differences in baseline social behavior during SA test, we evaluated the response of defeated mice in relation to same-strain controls. We divided the defeated mice into stress-susceptible and resilient groups, considering mice with SI ratios within 1 SD, or above, of the same-strain control mean as resilient (i.e., resembling the control mice), and those with the SI ratio below 1 SD from the mean as susceptible. We observed a significant difference in the distribution of susceptible and resilient mice between the strains (χ^2^ = 63.401, *p* = 1.102 × 10^−13^), with the B6 defeated mice being mostly resilient and the 129, BALB, and D2 mice mostly susceptible to stress (Fig. 1*B*). During the social target trial of the SA test, susceptible mice from all strains spent significantly less time in the IZ (Fig. 1*C*) and more time in the corners of the arena (Fig. 1*D*) than during the no-target trial.

10.1523/ENEURO.0166-18.2018.f1-1 Extended Data Figure 1-1 Exact *p* values for all behavioral tests. The test used for each parameter depicted in Figure 1*C–I* are outlined along with results of *post hoc* contrasts. D2: DBA/2NCrl strain; BALB: Balb/cAnNCrl; 129: 129S2/SvPasCrl; B6: C57BL/6NCrl. Download Figure 1-1, XLSX file.

We next determined how CSDS influences other behaviors. To assess locomotor behavior, we measured the distance moved during the no-target trial of the SA test (Fig. 1*E*) and the OF test (Fig. 1*F*). B6 and D2 defeated mice moved significantly less than control mice during both tests. We did not observe differences in distance traveled between defeated and control 129 or BALB mice. For the B6 strain, we also performed the EZM and FST to assess anxiety-like and despair behavior, respectively. Susceptible mice showed increased anxiety-like, but not despair behavior, compared to controls (Fig. 1*G*,*H*). To study metabolic effects of CSDS, we measured body weight before and after CSDS (Fig. 1*I*). All B6 mice gained weight (mixed ANOVA *post hoc* comparison before versus after CSDS: controls *p* = 9.98 × 10^−28^, resilient *p* = 2.21 × 10^−26^ and susceptible *p* = 6.23 × 10^−20^). BALB and 129 controls gained weight (*p* = 4.90 × 10^−5^ and *p* = 0.012, respectively), while the weight of the defeated mice of these strains did not change during CSDS. Both D2 resilient and susceptible mice lost weight (*p* = 0.004 and *p* = 2.53 × 10^−6^, respectively).

### Oligodendrocyte (OLG)-related genes are differentially expressed in response to stress

**Figure 2.**
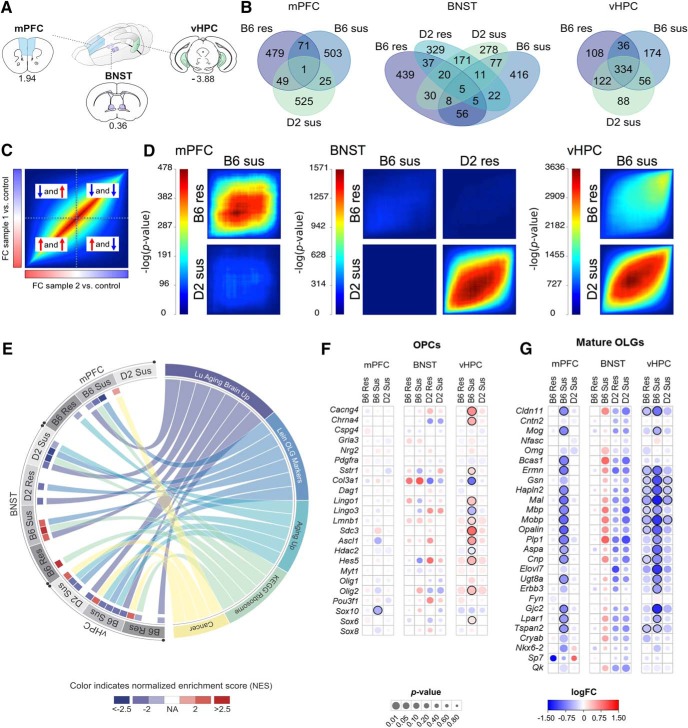
RNA-seq implicates OLG-related gene expression changes after CSDS. ***A***, Regions dissected for RNA-seq from D2 and B6 mice (for sample and RNA-seq details, see Extended Data Fig. 2-1). ***B***, Overlap of 300 top downregulated and 300 top upregulated genes between stressed and control mice, separately for each brain region (see Extended Data Fig. 2-2 for top 300 differentially expressed genes and Extended Data Fig. 2-3*A*,*B* for overlap separately for upregulated and downregulated genes). ***C***, Key to RRHO showing a hypothetical heatmap of two identical datasets (“FC sample 1 versus control” and “FC sample 2 versus control”). Following differential gene expression analysis, genes were ranked by their fold change (FC) and assigned to bins of 100 genes. Overlap of genes was then compared between each ranking matched bin of “sample 1 versus control” and “sample 2 versus control.” Heatmap color represents the significance of the overlap [-log10(*p*)] of genes between bins. Thus significant *p* values in the bottom-left corner indicate that the two datasets have shared upregulated genes, significant *p* values in the top-right corner indicate shared downregulated genes, and significant *p* values in the middle indicate genes not differentially expressed or with small FC. Significant *p* values in the top-left or bottom-right corner represent genes regulated in opposite directions between the two datasets. ***D***, RRHO shows significant similarity in the gene expression response to CSDS between resilient and susceptible mice within strains (B6 mPFC and vHPC, D2 BNST) and between susceptible mice of B6 and D2 strains (vHPC). Scale bar = –log(*p*) of rank classes (*n* = 100), for each brain region separately. ***E***, Circos plot showing the top five enriched gene sets overlapping between the brain regions in stress-resilient and susceptible mice compared to controls. Only normalized enrichment scores (NESs) achieving significance (*p_FDR_* < 0.05) are shown. A positive (or negative) NES for a given gene set indicates its overrepresentation at the top (or bottom, respectively) of the ranked list of upregulated (or downregulated, respectively) genes. See Extended Data Figures 2-4, 2-5 for GSEA and GO analyses, respectively; see Extended Data Figure 2-3*C* for expression FC for genes in the Lein OLG Markers gene set. ***F***, ***G***, Merged heat map showing the expression FC (logFC) and significance (*p*) of OPC-specific (***F***) and OLG-specific (***G***) genes. B6: C57BL/6NCrl strain; D2: DBA/2NCrl strain; Res: resilient; Sus: susceptible; mPFC: medial prefrontal cortex; BNST: bed nucleus of the stria terminalis; vHPC: ventral hippocampus; Lu Aging Brain Up: LU_AGING_BRAIN_UP; Lein OLG Markers: LEIN_OLIGODENDROCYTE_MARKERS; Aging Up: DEMAGALHAES_AGING_UP; KEGG Ribosome: KEGG_RIBOSOME; Cancer: GINESTIER_BREAST_CANCER_ZNF217_AMPLIFIED_DN.

To establish which biological pathways were affected by chronic stress, we conducted RNA-seq one week after CSDS in mPFC, vHPC, and BNST (Fig. 2*A* Extended Data Fig. 2-1). We selected the B6 and D2 strains for this analysis as they represented the phenotypic extremes in the proportions of susceptible and resilient mice. We were not able to analyze gene expression levels of resilient D2 mice for mPFC and vHPC due to low number of animals in this group. We always compared the stress-susceptible or resilient mice to the same-strain controls. We first determined the overlap of the top 300 up- and downregulated genes between the strains in each brain region (Fig. 2*B*; Extended Data Fig. 2-2), followed up by the RRHO analysis, which shows the overall similarity and direction of DE of all genes (Fig. 2*C*,*D*; Extended Data Fig. 2-3). In the mPFC, only 26 (2.2%) of the top differentially expressed genes between the susceptible versus control mice were shared between the B6 and D2 strains, and the RRHO analysis confirmed the highly divergent stress response of the two strains. In the BNST, the transcriptomic response of the two strains was marginally more similar as resilient B6 and D2 mice shared 67 (5.9%), and susceptible B6 and D2 mice shared 101 (9.2%) of the top genes. We detected the greatest overlap of the gene expression response between the strains in the vHPC, where B6- and D2 susceptible mice shared 390 (48.1%) of the top genes. Unlike in the mPFC or BNST, the stress effect was stronger than the strain effect in the vHPC. However, in the vHPC, several genes were downregulated both in the B6 susceptible and resilient mice.

10.1523/ENEURO.0166-18.2018.f2-1 Extended Data Figure 2-1 SI ratios, division of mice to phenotypic groups, library preparation and sequencing batches, and the number of analyzed RNA-seq reads. mPFC: medial prefrontal cortex; vHPC: ventral hippocampus; BNST: bed nucleus of the stria terminalis; B6: C57BL/6NCrl; D2: DBA/2NCrl. Download Figure 2-1, XLSX file.

10.1523/ENEURO.0166-18.2018.f2-2 Extended Data Figure 2-2 Top 300 upregulated and top 300 downregulated genes as ranked by -log10(*p*)*|logFC| for each comparison shown in Figure 2*B*. mPFC: medial prefrontal cortex; vHPC: ventral hippocampus; BNST: bed nucleus of stria terminalis; B6: C57BL/6NCrl; D2: DBA/2NCrl; Res: resilient; Sus: susceptible; Con: control. Download Figure 2-2, XLSX file.

**Figure 3.**
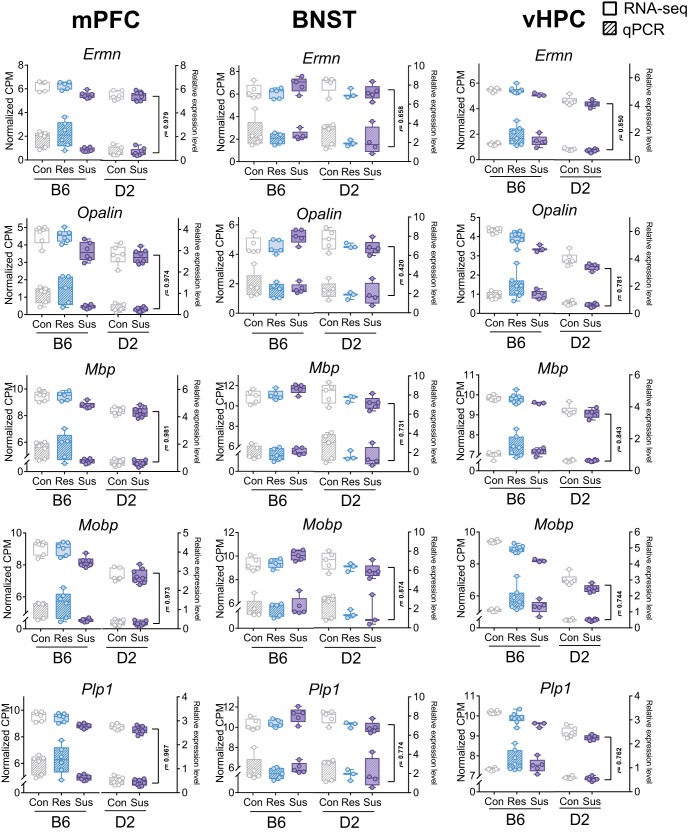
Strong correlation of gene expression levels of selected myelin-related genes determined by RNA-seq and q-RT-PCR. Box plots show the gene expression levels of five myelin-related genes (*Ermn*, *Opalin*, *Mbp*, *Mobp*, and *Plp1*) measured by RNA-seq (voom normalized number of reads, left *y*-axis, solid fill) and q-RT-PCR (right *y*-axis, striped fill). Pearson correlation coefficient (*r*) was calculated across the five (mPFC, vHPC) or six (BNST) group means. Box plots show distribution of values from min to max. mPFC: medial prefrontal cortex; vHPC: ventral hippocampus; BNST: bed nucleus of the stria terminalis; B6: C57BL/6NCrl: D2: DBA/2NCrl; Con: control; Res: resilient; Sus: susceptible.

10.1523/ENEURO.0166-18.2018.f2-3 Extended Data Figure 2-3 Overlap of the top differentially expressed and OLG-related genes after CSDS. *A*, ***B***, Overlap of the top 300 downregulated (***A***) and top 300 upregulated (***B***) genes between resilient versus control and susceptible versus control mice, separately for each brain region (Fig. 2*B*). ***C***, Merged heat map showing the expression fold change (FC) of resilient versus control and susceptible versus control groups. Genes belonging to the LEIN_OLIGODENDROCYTE_MARKERS gene set in the GSEA (Fig. 2*E*) are shown. FCs are shown only for genes with nominal *p* < 0.05. Genes which did not pass the cut-off are marked in grey (NA). mPFC: medial prefrontal cortex; vHPC: ventral hippocampus; BNST: bed nucleus of the stria terminalis; B6: C57BL/6NCrl; D2: DBA/2NCrl; Con: control; Res: resilient; Sus: susceptible. Download Figure 2-3, PDF file.

**Figure 4.**
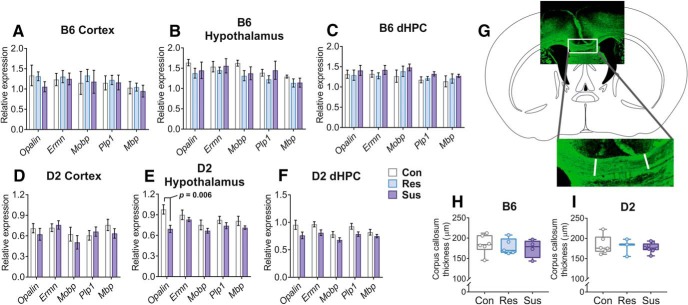
No generalized effects on OLG-related gene expression or corpus callosum thickness after CSDS. ***A–F***, Bar graphs showing the normalized expression levels of myelin-related genes in the hypothalamus and two brain regions not critically influenced by CSDS [cortex (without mPFC) and dHPC]. B6 and D2 mice were analyzed 6–8 d following CSDS. Myelin-related gene expression did not differ between phenotype groups in any of the brain regions of either strain. Only *Opalin* was expressed at a lower level in the hypothalamus of D2 susceptible mice compared to controls as shown by *post hoc* analysis. Mean ± 1 SEM is shown. ***G***, ***H***, CSDS does not affect corpus callosum thickness in B6 or D2 mice. Myelin visualized by anti-CNPase staining. Atlas outline modified from Franklin and Paxinos (2008); *n* = B6: Con = 6, Res = 5, Sus = 4; D2: Con = 6, Res = 3, Sus = 8. Error bars = min – max; D2: DBA/2NCrl strain; B6: C57BL/6NCrl strain; Con: control; Res: resilient; Sus: susceptible; dHPC: dorsal hippocampus.

10.1523/ENEURO.0166-18.2018.f2-4 Extended Data Figure 2-4 Pathway analysis results from GSEA for Figure 2*E*. Only *p_*FDR*_* < 0.05 are shown in the table. *p_*FDR*_*: a false discovery rate corrected *p* value; *p_*FWER*_*: familywise-error rate. mPFC: medial prefrontal cortex; vHPC: ventral hippocampus; BNST: bed nucleus of the stria terminalis; B6: C57BL/6NCrl; D2: DBA/2NCrl; Res: resilient; Sus: susceptible; Con: control. Download Figure 2-4, XLSX file.

**Figure 5.**
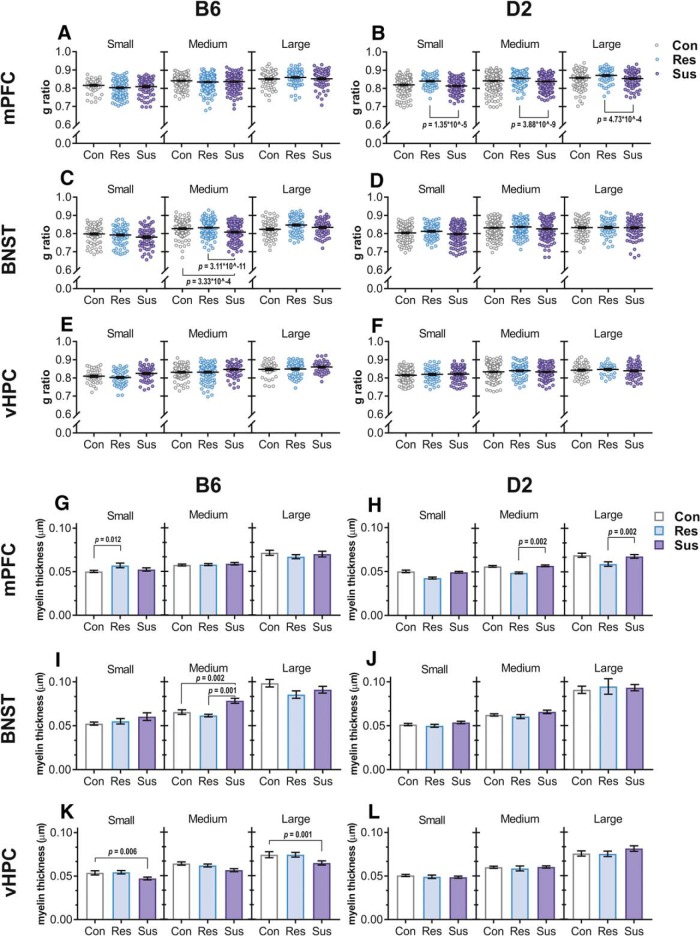
CSDS influences g ratio, myelin thickness, and axon diameter as measured by TEM. ***A–F***, Scatter plots of g ratio with mean indexed by a horizontal line. G ratio was lower in medium sized BNST axons of B6 susceptible mice compared to both control and resilient mice, and higher in all axon size categories in the mPFC of D2 resilient mice compared to susceptible mice. ***G–L***, Bar graphs of mean myelin thickness. Concurrently to g ratio measurements, B6 susceptible mice had thicker myelin on medium sized axons in the BNST compared to resilient or control mice, and D2 resilient mice had thinner myelin on medium and large axons in the mPFC compared to susceptible mice. Additionally, myelin was thicker in the small axons of the mPFC in B6 resilient mice compared to controls and thinner in the small and large axons of the vHPC in B6 susceptible mice compared to controls; *n* = B6: Con = 3, Res = 4, Sus = 3; D2: Con = 6, Res = 3, Sus = 5. Error bars ± 1 SEM. See Extended Data Figure 5-1 for schematics of dissected regions, Extended Data Figure 5-2 for analyses without division of axons into size categories, and Extended Data Figure 5-3 for size category division criteria. All nominal *p* values surviving Bonferroni correction are shown. D2: DBA/2NCrl; B6: C57BL/6NCrl; mPFC: medial prefrontal cortex; BNST: bed nucleus of the stria terminalis; vHPC: ventral hippocampus; Con: control; Res: resilient; Sus: susceptible.

10.1523/ENEURO.0166-18.2018.f2-5 Extended Data Figure 2-5 Significantly (*p* < 0.05) enriched GO terms within the top 300 upregulated and top 300 downregulated genes for each comparison, converging with results from GSEA (Fig. 2*E*). mPFC: medial prefrontal cortex; vHPC: ventral hippocampus; BNST: bed nucleus of the stria terminalis; B6: C57BL/6NCrl; D2: DBA/2NCrl; Res: resilient; Sus: susceptible; Con: control; BP: biological process; CC: cellular component; MF: molecular function. Download Figure 2-5, XLSX file.

To ask which biological pathways were affected by CSDS, we conducted GSEA and GO term enrichment analysis. GSEA showed significant enrichment of several gene sets (Extended Data Fig. 2-4), of which aging- and OLG-related sets were enriched in nearly all comparisons in all brain regions and both strains (Fig. 2*E*; Extended Data Fig. 2-3*C* ). Although functionally diverse, the genes included in the aging-related gene sets were significantly overrepresented in the “Lein OLG Markers” gene set (*p_FDR_* = 1.2 × 10^−16^). In accordance with GSEA, the most enriched GO terms were related to myelination and OLG development, in particular among the downregulated genes of B6 susceptible mice in the mPFC (Extended Data Fig. 2-5). These combined results from both enrichment analyses prompted us to further investigate OLG-related genes.

Mature OLGs develop from OPCs, which persist even in the adult brain as committed precursors. We asked whether either OPC or OLG cell populations dominantly contributed to the observed DE. The transcriptomic signature associated with CSDS was stronger in the mature OLG markers (Fig. 2*G*) than in the OPC markers (Fig. 2*F*). To validate the RNA-seq findings, we analyzed *Opalin*, *Ermn*, *Mobp*, *Plp1*, and *Mbp* gene expression levels with q-RT-PCR in mPFC, BNST, and vHPC and found high correlation with RNA-seq and q-RT-PCR measurements of these myelination-related genes (mean *r* = 0.82; Fig. 3).

### Myelin-related variation after stress is not observed globally in the brain

To test whether differences in myelin-related gene expression were observed across the brain, we measured expression levels of *Opalin, Ermn, Mobp, Plp1*, and *Mbp* in the whole cortex (lacking the mPFC), hypothalamus, and dorsal hippocampus (dHPC) by q-RT-PCR. Using mixed ANOVA we determined that there was no significant main effect by the group (control, resilient or susceptible) on myelin-related gene expression in any of the brain regions of either strain. While *Opalin* expression was lower in D2 susceptible mice compared to controls in the hypothalamus as determined by *post hoc* comparison (*p* = 0.006), the expression levels of the other genes did not differ between the stressed and control mice in any region (Fig. 4*A–F*). We also measured the thickness of the corpus callosum in brain sections stained with a myelin-binding antibody (anti-CNPase) (Fig. 4*G*). We observed no differences in stress-susceptible or resilient mice compared to controls (Fig. 4*H*,*I*).

### Myelin thickness and g ratio differ in brain region and genetic background-dependent manner

To determine whether CSDS affects myelination, we conducted TEM of myelinated axons in mPFC, BNST, and vHPC. In addition to analyzing axons of different diameters within each brain region and stress group together (Extended Data Fig. 5-2), we divided the axons to three size groups given that different types of neuronal projections may differ in axon diameter (Fig. 5; Extended Data Fig. 5-3). We discovered several brain region- and strain-specific differences between stress groups in g ratio, i.e., the ratio of the inner axonal diameter to the total outer diameter, myelin thickness, and axon diameter. Overall, D2 resilient mice had higher g ratio and thinner myelin in the mPFC compared to susceptible mice. B6 susceptible mice had thinner myelin in the vHPC compared to controls and thicker myelin in the BNST compared to resilient mice (Extended Data Fig. 5-2). In addition to these general findings, we found significant differences between stress susceptible or resilient mice compared to controls in axons of certain diameter (Fig. 5). We also observed modestly but significantly smaller axon diameter (without the myelin sheath) in the mPFC of D2 susceptible mice compared to controls (Extended Data Fig. 5-2).

10.1523/ENEURO.0166-18.2018.f5-1 Extended Data Figure 5-1 Regions dissected for TEM. Purple shaded squares outline the dissected samples from 200-µm sections. Atlas outlines are based on Franklin and Paxinos (2008), and the distance from the midline (sagittal) in millimeters is shown below each image. Download Figure 5-1, PDF file.

10.1523/ENEURO.0166-18.2018.f5-2 Extended Data Figure 5-2 Effect of CSDS on g ratio (***A***) and myelin thickness (***B***) when comparing all axons without division into axon size categories (for results with division into small, medium, and large axon size categories, see Fig. 5) and on axon diameter (***C***). ***D***, Representative TEM images for each group, scale bar = 0.5 µm. Error bars ± 1 SEM. All nominal *p* values surviving Bonferroni correction against α = 0.0167 are shown. Download Figure 5-2, PDF file.

10.1523/ENEURO.0166-18.2018.f5-3 Extended Data Figure 5-3 Details of axons assessed by TEM (depicted in Fig. 5). Calculation of axon diameter category boundaries (small, medium, large) in each brain region, and the number of animals and axons per category and group. mPFC: medial prefrontal cortex; vHPC: ventral hippocampus; BNST: bed nucleus of the stria terminalis; B6: C57BL/6NCrl; D2: DBA/2NCrl; Res: resilient; Sus: susceptible; Con: control. Download Figure 5-3, XLSX file.

## Discussion

We established that the behavioral and brain transcriptomic responses to chronic psychosocial stress are genetically controlled. We discovered that BALB, 129, and D2 mice were more susceptible to chronic stress than B6 mice. We demonstrated, by unbiased RNA-seq, that after CSDS the most significantly affected gene sets and biological pathways in the mPFC, vHPC, and BNST were related to myelination. Consistently, we observed significant brain region and strain-dependent differences in myelin thickness after stress. Neither myelin gene expression in other cortical regions or the dHPC, nor corpus callosum thickness, differed between stressed and control mice, suggesting no overall white matter changes due to CSDS.

We first established the CSDS paradigm in four inbred mouse strains and demonstrated that genetic factors control their adaptive behavior to stress. Consistently with prior findings (Razzoli et al., 2011a; Savignac et al., 2011), BALB mice were more sensitive to CSDS-induced SA than B6 mice. Additionally, innately anxious D2 and 129 mice were also highly susceptible to CSDS, suggesting that anxious strains may in general be stress-sensitive. CSDS did not affect locomotor activity of 129 or BALB mice, but both resilient and susceptible mice failed to gain weight during the defeat period. By contrast, the defeated D2 mice had lower locomotor activity than controls, and they lost weight during defeat. Differently to the other strains, the defeated B6 mice had lower locomotor activity compared to controls but they gained weight during defeat, similarly to controls. We also investigated other behaviors in this strain, and found that the stress-susceptible mice had increased anxiety-like behavior, but no difference in despair behavior, as in previous studies (Krishnan et al., 2007; Razzoli et al., 2011a). We acknowledge that due to limitations of our animal facility, all mice were brought into the experimental room together before behavioral testing, where they stayed behind a screen during the testing of other animals, possibly introducing confounding factors. Overall, our results suggest that the studied strains use different coping strategies to stress, and that such behavior has a strong genetic basis.

In addition to different behavioral responses to CSDS, we observed large differences in the brain transcriptomic response to stress between B6 and D2 strains. mPFC and BNST expression patterns were more similar within strains, between the resilient and susceptible mice, than between susceptible or resilient mice of different strains. However, in vHPC, the transcriptomic response of B6 and D2 susceptible mice was highly similar. This result may reflect the different organization and roles of these three brain regions in processing stress-related signals. It may be that the vHPC relays primary information regarding stress, while the BNST and mPFC may have more interpreting and processing roles, and therefore little genetic variation is tolerated within the hippocampal stress response. vHPC gene expression is influenced by glucocorticoids through glucocorticoid receptors, which are strongly expressed in the hippocampus (Ahima and Harlan, 1990; Ahima et al., 1991; Gray et al., 2017). BNST receives projections from several amygdalar nuclei and mediates anxiety-related information to hypothalamic and brainstem targets (Davis et al., 2010). The mPFC influences stress-associated social behavior by evaluation of perceived threats, top-down control of goal-directed behavior (Carlén, 2017), and emotion regulation (Etkin et al., 2015). These differences reflected on the transcriptomic response to stress may contribute to the distinct coping strategies of the strains.

We found significant enrichment of OLG- and aging-related genes within the differentially expressed genes in both strains and all brain regions. The highly significant overlap between both of these gene sets suggests similar changes in OLG-related gene expression may be associated with both aging and chronic psychosocial stress. This OLG-related signal was derived mostly from genes expressed in mature OLGs, which are the myelin-producing cells in the central nervous system. Myelin plasticity, a response of OLGs to neuronal activity (Gautier et al., 2015; Koudelka et al., 2016; Purger et al., 2016; Mitew et al., 2018), is retained in adulthood (Bengtsson et al., 2005; McKenzie et al., 2014). Downregulation of OLG-related genes was especially pronounced in the vHPC, and also in the mPFC of B6 susceptible mice. On the structural level, vHPC myelin sheaths of B6 susceptible mice were thinner than in control mice, concurring with the downregulation of myelin-related genes. Interestingly, in the BNST, B6 susceptible mice had thicker myelin sheaths and smaller g ratio compared to both resilient and control mice, also concurring with the gene expression patterns. D2 susceptible mice had an opposite gene expression pattern in the BNST, which, however, was not supported by changes in myelin sheath thickness or g ratio. Opposite gene expression changes in different inbred mouse strains in response to stress have previously been observed in other models, but the underlying mechanisms are not fully understood (Mozhui et al., 2010; Malki et al., 2015). In mice, various stressors have diverse effects on myelination, likely reflecting involvement of distinct neural processes, developmental stage at the time of stress exposure, and duration of stress. Early life stress affects myelin-related gene expression in the mPFC (Bordner et al., 2011; Makinodan et al., 2012), and myelination of the amygdala (Ono et al., 2008). In adult mice, social isolation (Liu et al., 2012) and 14-d CSDS in susceptible mice (Lehmann et al., 2017) reduce myelin-related gene expression and myelination within the frontal cortex. Intermittent social defeat stress leads to reduced MBP-stained myelin area in the mPFC (Zhang et al., 2016). Corpus callosum myelination is decreased after chronic restraint stress (Choi et al., 2017). Also chronic variable stress induces temporally variable myelin-related gene expression changes, and these changes were regionally selective in the mPFC, nucleus accumbens, and corpus callosum (Liu et al., 2018). Therefore, it is likely that alterations in gene expression and physical parameters of the myelin sheaths occur dynamically over days and weeks (Montesinos et al., 2015; Almeida and Lyons, 2017), possibly explaining thicker myelin in B6 resilient mice in the mPFC, without differences in myelin-related gene expression compared to controls. Overall, our results concur with earlier studies and suggest that stress does not simply cause widespread downregulation of myelin-related genes or myelin loss, but that stress-influenced myelin-plasticity involves specific stress-associated brain circuits.

Our strategy to divide the stressed animals to susceptible and resilient groups allowed identification of specific patterns of myelination-related differences between these groups. We observed that chronic stress associates with thicker myelin in the susceptible (in the BNST of B6 mice) and thinner myelin in resilient mice (D2 mPFC). No prior studies exist, to our knowledge, on resilience-related myelin thinning. Although DTI measures are only considered proxies for myelination, in humans, baseline fractional anisotropy has been demonstrated to correlate positively with state anxiety, but following exposure to a traumatic event, correlation is negative (Sekiguchi et al., 2014). Furthermore, increased fractional anisotropy, suggestive of enhanced integrity, has been reported in the cingulate gray matter of panic disorder patients (Han et al., 2008). Also, the number of mature OLGs in the ventromedial PFC of depressed subjects with childhood abuse is increased (Tanti et al., 2017). Our results suggest that these complex patterns are strongly modulated by genetic factors.

Although myelination studies on human anxiety disorders and chronic stress are scarce, major depression has been consistently associated with white matter disruptions (Wang et al., 2014). Depressed suicide completers with childhood abuse have impaired myelin-related gene expression and reduced myelin thickness in the anterior cingulate cortex (Lutz et al., 2017). Interestingly, this effect was only seen in small caliber axons, similarly to our finding of mPFC myelin thickness being larger in B6 resilient mice compared to controls. Axons of different diameter may represent specific types of neurons (Perge et al., 2012) and therefore be differentially vulnerable to the effects of stress. For example, in the macaque cortex, axons which project longer distance from the point of origin have larger diameters than those projecting to proximal targets (Innocenti et al., 2014). Based on the axon diameters measured by TEM, it is not possible to infer the type of neurons affected in our experiment but this question could be addressed with immuno-electron microscopy.

How may structural changes in myelin affect behavior? Myelin thickness is a key component in determining axonal conduction speed, thereby influencing circuit function. Myelin deficient rats have altered conduction time and lower average synchrony levels in the cerebellum (Lang and Rosenbluth, 2003). Mice have increased synchronous activity between the vHPC and mPFC in an anxiogenic environment (Adhikari et al., 2010). Furthermore, in primates, increased power and phase synchrony in the theta range has been detected in the amygdala-prefrontal circuit during aversive conditioning (Taub et al., 2018). Altered synchrony has also been recorded in human psychiatric disorders (Schulman et al., 2011; Leuchter et al., 2015). Genetic factors affect synchronous oscillations during sleep in the inbred mouse strains (Franken et al., 1998; Tafti et al., 2003). The role of brain oscillations in myelination and psychiatric phenotypes are still poorly understood, but myelin plasticity may be a mechanism that allows regulation of axonal conduction and spatial connectivity.

Overall, our unbiased brain gene expression analysis suggests that myelin plasticity is among the most significant responses to chronic psychosocial stress. Our strategy to divide the mice into stress-susceptible and resilient groups allowed us to demonstrate significant differences in myelination-related gene expression and myelin thickness between these groups. Furthermore, variable myelin plasticity across brain regions suggests that chronic stress has localized effects on myelination. Importantly, by using two different inbred mouse strains we demonstrated that stress-induced myelin plasticity is genetically controlled. Identification of the genetic regulators of the myelin response will provide mechanistic insight into the molecular basis of stress-induced anxiety, a critical step in developing targeted therapy for anxiety disorders.

## Synthesis

Reviewing Editor: Tallie Z. Baram, University of California-Irvine Decisions are customarily a result of the Reviewing Editor and the peer reviewers coming together and discussing their recommendations until a consensus is reached. When revisions are invited, a fact-based synthesis statement explaining their decision and outlining what is needed to prepare a revision will be listed below. The following reviewer(s) agreed to reveal their identity: Carmen Sandi. Note: If this manuscript was transferred from JNeurosci and a decision was made to accept the manuscript without peer review, a brief statement to this effect will instead be what is listed below.

Both reviewers felt that this manuscript provides valuable comparative data regarding different mouse strains' gene expression response to stress in multiple brain regions and identifies myelin/oligodendrocyte and aging pathways as particularly important. One of the reviewers, who had seen the original version, appreciated the authors' efforts in improving the paper. Both reviewers and i believe that the work will be a nice addition to our body of knowledge, pending improvement of several remaining issues.

These are enumerated below:

a. General issues

-Why did the authors only focus on oligodendrogenesis/myelin gene sets? Ageing gene sets were also highly enriched. Some discussion of the aging pathway would be a very useful addition in discussion, if not in results too.

--Regarding the electron microscopy data, was each axon treated as a single “n”? The number of data points in Fig. 4 suggest this is so. This seems like an inappropriate analysis to to increase power if so--in extended data, looks like the data points came from 3-6 animals depending on group. How many axons per animal? Is this what is referred to in lines 309-313 of methods? Please clarify. It would be more appropriate to have summed data for each animal and compare across animals, not across cells. Similarly, the EM data should be compared using repeated measures for the different caliber axons.

--One main concern is that some data analysis and presentation is unclear. In general, the figure captions are lacking in crucial detail and summary descriptions of the effects observed. Adding those details in every figure caption would help (e.g., Fig 1b: Susceptible B6 animals showed...) Specific comments for figures/data follow.

Figure 1: Panel B--what is Y axis measure? Not described in caption. Social avoidance % should be lower in susceptible than resilient animals, correct? If a lower score means more avoidance, that needs to be spelled out in caption. What is pie chart with % showing? Describe in caption. Also, little colored mouse cartoons aren't adding any useful informationand should be removed.

Figure 1C-D are way too small to read easily--please enlarge.

Figure 2--Clarification on how panel C helps read panel D would be useful.

Figure 2 or 3 should contain supplemental figure 2.6--this data is far too crucial to be in supplemental. '

Supplemental Figure 2-- figure caption says correlations performed with all 6 groups, but for both VHPC and PFC, it is only 5 groups, correct? Also, I would prefer to see this data represented as scatterplots for each gene instead of boxplots to see the overall shape of the data and coherence across different groups. Also, for Fig 2.6, it would be nice to see the qPCR data analyzed on its own for main effect of phenotype, not just correlated with RNAseq data, though the correlations are excellent to have and should of course remain a focus of the figure. These data come from the same samples, correct?

Figure 3-- Panels A-F very small--please enlarge. Please clarify how data was analyzed. It seems inappropriate to do a single t-test for every gene in D2 and a one-way ANOVA in B6s--what if analyzed with repeated measures? Might then see a main effect of resilient/susceptible phenotype? Figure 3G--it would be far better to have the low-magnification schematic replaced by a low-magnification image of the actual myelin staining.

Discussion-- Still could use some expansion of speculation about different caliber neurons and changes in myelination after stress. Which axons would potentially be affected--e.g., which neurons in these regions have smaller diameter axons?

Minor concerns:

Line 357-8 sentence needs clarification--confusing. “However, B6 susceptible and resilient mice did share especially a number of downregulated genes.”

Line 322- same-stain should be same-strain

b. Statistical issues:

FIG 3: Please clarify how data was analyzed. It seems inappropriate to do a single t-test for every gene in D2 and a one-way ANOVA in B6s--what if analyzed with repeated measures? Might then see a main effect of resilient/susceptible phenotype? Figure 3G--it would be far better to have the low-magnification schematic replaced by a low-magnification image of the actual myelin staining.

FIG 4: Regarding the electron microscopy data, was each axon treated as a single “n”? The number of data points in Fig. 4 suggest this is so. This seems like an inappropriate analysis to to increase power if so--in extended data, looks like the data points came from 3-6 animals depending on group. How many axons per animal? Is this what is referred to in lines 309-313 of methods? Please clarify. It would be more appropriate to have summed data for each animal and compare across animals, not across cells. Similarly, the EM data should be compared using repeated measures for the different caliber axons.

c. Significant methodological issue. please acknowledge this caveat in the discussion.

This reviewer would like to point to the authors that when all animals are taken together to the experimental room and stay there throughout behavioral testing, there are many confounding factors which one cannot discard and that, therefore, it is advisable to bring experimental animals one-by-one and return them to the housing room right after testing. This ensures a better standardization of the procedures in terms of time animals are in the testing room before testing, possible vocalizations from testing and/or waiting animals that can perturb ongoing behavior or behavioral states, etc, etc.

## References

[B1] Adhikari A, Topiwala MA, Gordon JA (2010) Synchronized activity between the ventral hippocampus and the medial prefrontal cortex during anxiety. Neuron 65:257–269. 10.1016/j.neuron.2009.12.002 20152131 PMC2822726

[B2] Ahima RS, Harlan RE (1990) Charting of type II glucocorticoid receptor-like immunoreactivity in the rat central nervous system. Neuroscience 39:579–604. 1711170 10.1016/0306-4522(90)90244-x

[B3] Ahima RS, Krozowski Z, Harlan R (1991) Type I corticosteroid receptor-like immunoreactivity in the rat CNS: distribution and regulation by corticosteroids. J Comp Neur 313:522–538. 10.1002/cne.903130312 1770174

[B4] Alexa A, Rahnenführer J, Lengauer T (2006) Improved scoring of functional groups from gene expression data by decorrelating GO graph structure. Bioinformatics 22:1600–1607. 10.1093/bioinformatics/btl140 16606683

[B5] Almeida RG, Lyons DA (2017) On myelinated axon plasticity and neuronal circuit formation and function. J Neurosci 37:10023–10034. 10.1523/JNEUROSCI.3185-16.2017 29046438 PMC6596541

[B6] Anders S, Pyl PT, Huber W (2015) HTSeq–a Python framework to work with high-throughput sequencing data. Bioinformatics 31:166–169. 10.1093/bioinformatics/btu638 25260700 PMC4287950

[B7] Avgustinovich DF, Kovalenko IL, Kudryavtseva NN (2005) A model of anxious depression: persistence of behavioral pathology. Neurosci Behav Physiol 35:917–924. 10.1007/s11055-005-0146-6 16270173

[B8] Bengtsson SL, Nagy Z, Skare S, Forsman L, Forssberg H, Ullén F (2005) Extensive piano practicing has regionally specific effects on white matter development. Nat Neurosci 8:1148–1150. 10.1038/nn1516 16116456

[B9] Benjamini Y, Hochberg Y (1995) Controlling the false discovery rate: a practical and powerful approach to multiple testing. J R Stat Soc Series B Stat Methodol 57:289–300.

[B10] Bordner KA, George ED, Carlyle BC, Duque A, Kitchen RR, Lam TT, Colangelo CM, Stone KL, Abbott TB, Mane SM, Nairn AC, Simen AA (2011) Functional genomic and proteomic analysis reveals disruption of myelin-related genes and translation in a mouse model of early life neglect. Front Psychiatry 2:18. 10.3389/fpsyt.2011.00018 21629843 PMC3098717

[B11] Calhoon GG, Tye KM (2015) Resolving the neural circuits of anxiety. Nat Neurosci 18:1394–1404. 10.1038/nn.4101 26404714 PMC7575249

[B12] Carlén M (2017) What constitutes the prefrontal cortex? Science 358:478–482. 10.1126/science.aan8868 29074767

[B13] Choi MH, Na JE, Yoon YR, Lee HJ, Yoon S, Rhyu IJ, Baik JH (2017) Role of dopamine D2 receptor in stress-induced myelin loss. Sci Rep 7:11654. 10.1038/s41598-017-10173-9 28912499 PMC5599541

[B14] Davis M, Walker DL, Miles L, Grillon C (2010) Phasic vs sustained fear in rats and humans: role of the extended amygdala in fear vs anxiety. Neuropsychopharmacology 35:105–135. 10.1038/npp.2009.109 19693004 PMC2795099

[B15] Dobin A, Davis CA, Schlesinger F, Drenkow J, Zaleski C, Jha S, Batut P, Chaisson M, Gingeras TR (2013) STAR: ultrafast universal RNA-seq aligner. Bioinformatics 29:15–21. 10.1093/bioinformatics/bts635 23104886 PMC3530905

[B16] Donner J, Sipilä T, Ripatti S, Kananen L, Chen X, Kendler KS, Lönnqvist J, Pirkola S, Hettema JM, Hovatta I (2012) Support for involvement of glutamate decarboxylase 1 and neuropeptide Y in anxiety susceptibility. Am J Med Genet B Neuropsychiatr Genet 159B:316–327. 10.1002/ajmg.b.32029 22328461

[B17] Ducottet C, Belzung C (2005) Correlations between behaviours in the elevated plus-maze and sensitivity to unpredictable subchronic mild stress: evidence from inbred strains of mice. Behav Brain Res 156:153–162. 10.1016/j.bbr.2004.05.018 15474660

[B18] Etkin A, Büchel C, Gross JJ (2015) The neural bases of emotion regulation. Nat Rev Neurosci 16:693–700. 10.1038/nrn4044 26481098

[B19] Franken P, Malafosse A, Tafti M (1998) Genetic variation in EEG activity during sleep in inbred mice. Am J Physiol 275:R1127–R1137. 9756543 10.1152/ajpregu.1998.275.4.R1127

[B20] Franklin KBJ, Paxinos G (2008) The mouse brain in stereotaxic coordinates, Ed 3 New York, NY: Academic Press.

[B21] Garakani A, Murrough JW, Charney JD, Bremner JD (2009) The neurobiology of anxiety disorders In: Neurobiology of mental illness, Ed 4 (Charney DS, Sklar P, Buxbaum J, Nestler EJ, eds), pp 655–690. Oxford; New York: Oxford University Press.

[B22] Gautier HO, Evans KA, Volbracht K, James R, Sitnikov S, Lundgaard I, James F, Lao-Peregrin C, Reynolds R, Franklin RJ, Káradóttir RT (2015) Neuronal activity regulates remyelination via glutamate signalling to oligodendrocyte progenitors. Nat Commun 6:8518. 10.1038/ncomms9518 26439639 PMC4600759

[B23] Gentleman R, Carey V, Huber W, Hahne F (2017) Genefilter: methods for filtering genes from high-throughput experiments. R package version 1.60.0.

[B24] Golden SA, Covington HE 3rd, Berton O, Russo SJ (2011) A standardized protocol for repeated social defeat stress in mice. Nat Protoc 6:1183–1191. 10.1038/nprot.2011.361 21799487 PMC3220278

[B25] Gray JD, Kogan JF, Marrocco J, McEwen BS (2017) Genomic and epigenomic mechanisms of glucocorticoids in the brain. Nat Rev Endocrinol 13:661–673. 10.1038/nrendo.2017.97 28862266

[B26] Haarman BC, Riemersma-Van der Lek RF, Burger H, Netkova M, Drexhage RC, Bootsman F, Mesman E, Hillegers MH, Spijker AT, Hoencamp E, Drexhage HA, Nolen WA (2014) Relationship between clinical features and inflammation-related monocyte gene expression in bipolar disorder - towards a better understanding of psychoimmunological interactions. Bipolar Disord 16:137–150. 10.1111/bdi.12142 24286609

[B27] Hammels C, Pishva E, De Vry J, van den Hove DL, Prickaerts J, van Winkel R, Selten JP, Lesch KP, Daskalakis NP, Steinbusch HW, van Os J, Kenis G, Rutten BP (2015) Defeat stress in rodents: from behavior to molecules. Neurosci Biobehav Rev 59:111–140. 10.1016/j.neubiorev.2015.10.006 26475995

[B28] Han DH, Renshaw PF, Dager SR, Chung A, Hwang J, Daniels MA, Lee YS, Lyoo IK (2008) Altered cingulate white matter connectivity in panic disorder patients. J Psychiatr Res 42:399–407. 10.1016/j.jpsychires.2007.03.002 17482647

[B29] Han MH, Nestler EJ (2017) Neural substrates of depression and resilience. Neurotherapeutics 14:677–686. 10.1007/s13311-017-0527-x 28397115 PMC5509625

[B30] Hanley JA, Negassa A, Edwardes MD, Forrester JE (2003) Statistical analysis of correlated data using generalized estimating equations: an orientation. Am J Epidemiol 157:364–375. 12578807 10.1093/aje/kwf215

[B31] Hettema JM, Neale MC, Kendler KS (2001) A review and meta-analysis of the genetic epidemiology of anxiety disorders. Am J Psychiatry 158:1568–1578. 10.1176/appi.ajp.158.10.1568 11578982

[B32] Hovatta I, Barlow C (2008) Molecular genetics of anxiety in mice and men. Ann Med 40:92–109. 10.1080/07853890701747096 18293140

[B33] Hovatta I, Tennant RS, Helton R, Marr RA, Singer O, Redwine JM, Ellison JA, Schadt EE, Verma IM, Lockhart DJ, Barlow C (2005) Glyoxalase 1 and glutathione reductase 1 regulate anxiety in mice. Nature 438:662–666. 10.1038/nature04250 16244648

[B34] Howlett JR, Stein MB (2016) Prevention of trauma and stressor-related disorders: a review. Neuropsychopharmacology 41:357–369. 10.1038/npp.2015.261 26315508 PMC4677144

[B35] Innocenti GM, Vercelli A, Caminiti R (2014) The diameter of cortical axons depends both on the area of origin and target. Cereb Cortex 24:2178–2188. 10.1093/cercor/bht070 23529006

[B36] Johnson WE, Li C, Rabinovic A (2007) Adjusting batch effects in microarray expression data using empirical Bayes methods. Biostatistics 8:118–127. 10.1093/biostatistics/kxj037 16632515

[B37] Kemeny ME, Schedlowski M (2007) Understanding the interaction between psychosocial stress and immune-related diseases: a stepwise progression. Brain Behav Immun 21:1009–1018. 10.1016/j.bbi.2007.07.010 17889502

[B38] Koudelka S, Voas MG, Almeida RG, Baraban M, Soetaert J, Meyer MP, Talbot WS, Lyons DA (2016) Individual neuronal subtypes exhibit diversity in CNS myelination mediated by synaptic vesicle release. Curr Biol 26:1447–1455. 10.1016/j.cub.2016.03.070 27161502 PMC4906267

[B39] Krishnan V, Han MH, Graham DL, Berton O, Renthal W, Russo SJ, Laplant Q, Graham A, Lutter M, Lagace DC, Ghose S, Reister R, Tannous P, Green TA, Neve RL, Chakravarty S, Kumar A, Eisch AJ, Self DW, Lee FS, et al. (2007) Molecular adaptations underlying susceptibility and resistance to social defeat in brain reward regions. Cell 131:391–404. 10.1016/j.cell.2007.09.018 17956738

[B40] Krzywinski M, Schein J, Birol I, Connors J, Gascoyne R, Horsman D, Jones SJ, Marra MA (2009) Circos: an information aesthetic for comparative genomics. Genome Res 19:1639–1645. 10.1101/gr.092759.109 19541911 PMC2752132

[B41] Laine MA, Sokolowska E, Dudek M, Callan SA, Hyytiä P, Hovatta I (2017) Brain activation induced by chronic psychosocial stress in mice. Sci Rep 7:15061. 10.1038/s41598-017-15422-5 29118417 PMC5678090

[B42] Lang EJ, Rosenbluth J (2003) Role of myelination in the development of a uniform olivocerebellar conduction time. J Neurophysiol 89:2259–2270. 10.1152/jn.00922.2002 12611949

[B43] Law CW, Chen Y, Shi W, Smyth GK (2014) voom: precision weights unlock linear model analysis tools for RNA-seq read counts. Genome Biol 15:R29. 10.1186/gb-2014-15-2-r29 24485249 PMC4053721

[B44] Lehmann ML, Weigel TK, Elkahloun AG, Herkenham M (2017) Chronic social defeat reduces myelination in the mouse medial prefrontal cortex. Sci Rep 7:46548. 10.1038/srep46548 28418035 PMC5394533

[B45] Leuchter AF, Hunter AM, Krantz DE, Cook IA (2015) Rhythms and blues: modulation of oscillatory synchrony and the mechanism of action of antidepressant treatments. Ann NY Acad Sci 1344:78–91. 10.1111/nyas.12742 25809789 PMC4412810

[B46] Liu J, Dietz K, DeLoyht JM, Pedre X, Kelkar D, Kaur J, Vialou V, Lobo MK, Dietz DM, Nestler EJ, Dupree J, Casaccia P (2012) Impaired adult myelination in the prefrontal cortex of socially isolated mice. Nat Neurosci 15:1621–1623. 10.1038/nn.3263 23143512 PMC3729624

[B47] Liu J, Dietz K, Hodes GE, Russo SJ, Casaccia P (2018) Widespread transcriptional alternations in oligodendrocytes in the adult mouse brain following chronic stress. Dev Neurobiol 78:152–162. 10.1002/dneu.22533 28884925 PMC5773375

[B48] Lutz PE, Tanti A, Gasecka A, Barnett-Burns S, Kim JJ, Zhou Y, Chen GG, Wakid M, Shaw M, Almeida D, Chay MA, Yang J, Larivière V, M'Boutchou MN, van Kempen LC, Yerko V, Prud'homme J, Davoli MA, Vaillancourt K, Théroux JF, et al. (2017) Association of a history of child abuse with impaired myelination in the anterior cingulate cortex: convergent epigenetic, transcriptional, and morphological evidence. Am J Psychiatry 174:1185–1194. 10.1176/appi.ajp.2017.16111286 28750583

[B49] Makinodan M, Rosen KM, Ito S, Corfas G (2012) A critical period for social experience-dependent oligodendrocyte maturation and myelination. Science 337:1357–1360. 10.1126/science.1220845 22984073 PMC4165613

[B50] Malki K, Mineur YS, Tosto MG, Campbell J, Karia P, Jumabhoy I, Sluyter F, Crusio WE, Schalkwyk LC (2015) Pervasive and opposing effects of unpredictable chronic mild stress (UCMS) on hippocampal gene expression in BALB/cJ and C57BL/6J mouse strains. BMC Genomics 16:262. 10.1186/s12864-015-1431-6 25879669 PMC4412144

[B51] Marques S, Zeisel A, Codeluppi S, van Bruggen D, Mendanha Falcão A, Xiao L, Li H, Häring M, Hochgerner H, Romanov RA, Gyllborg D, Muñoz Manchado A, La Manno G, Lönnerberg P, Floriddia EM, Rezayee F, Ernfors P, Arenas E, Hjerling-Leffler J, et al. (2016) Oligodendrocyte heterogeneity in the mouse juvenile and adult central nervous system. Science 352:1326–1329. 10.1126/science.aaf6463 27284195 PMC5221728

[B52] McKenzie IA, Ohayon D, Li H, de Faria JP, Emery B, Tohyama K, Richardson WD (2014) Motor skill learning requires active central myelination. Science 346:318–322. 10.1126/science.1254960 25324381 PMC6324726

[B53] Millstein RA, Holmes A (2007) Effects of repeated maternal separation on anxiety- and depression-related phenotypes in different mouse strains. Neurosci Biobehav Rev 31:3–17. 10.1016/j.neubiorev.2006.05.003 16950513

[B54] Mitew S, Gobius I, Fenlon LR, McDougall SJ, Hawkes D, Xing YL, Bujalka H, Gundlach AL, Richards LJ, Kilpatrick TJ, Merson TD, Emery B (2018) Pharmacogenetic stimulation of neuronal activity increases myelination in an axon-specific manner. Nat Commun 9:306. 10.1038/s41467-017-02719-2 29358753 PMC5778130

[B55] Moffitt TE, Caspi A, Harrington H, Milne BJ, Melchior M, Goldberg D, Poulton R (2007) Generalized anxiety disorder and depression: childhood risk factors in a birth cohort followed to age 32. Psychol Med 37:441–452. 10.1017/S0033291706009640 17201999

[B56] Montesinos J, Pascual M, Pla A, Maldonado C, Rodríguez-Arias M, Miñarro J, Guerri C (2015) TLR4 elimination prevents synaptic and myelin alterations and long-term cognitive dysfunctions in adolescent mice with intermittent ethanol treatment. Brain Behav Immun 45:233–244. 10.1016/j.bbi.2014.11.015 25486089

[B57] Mootha VK, Lindgren CM, Eriksson KF, Subramanian A, Sihag S, Lehar J, Puigserver P, Carlsson E, Ridderstråle M, Laurila E, Houstis N, Daly MJ, Patterson N, Mesirov JP, Golub TR, Tamayo P, Spiegelman B, Lander ES, Hirschhorn JN, Altshuler D, et al. (2003) PGC-1alpha-responsive genes involved in oxidative phosphorylation are coordinately downregulated in human diabetes. Nat Genet 34:267–273. 10.1038/ng1180 12808457

[B58] Mozhui K, Karlsson RM, Kash TL, Ihne J, Norcross M, Patel S, Farrell MR, Hill EE, Graybeal C, Martin KP, Camp M, Fitzgerald PJ, Ciobanu DC, Sprengel R, Mishina M, Wellman CL, Winder DG, Williams RW, Holmes A (2010) Strain differences in stress responsivity are associated with divergent amygdala gene expression and glutamate-mediated neuronal excitability. J Neurosci 30:5357–5367. 10.1523/JNEUROSCI.5017-09.2010 20392957 PMC2866495

[B59] Nasca C, Bigio B, Zelli D, Nicoletti F, McEwen BS (2015) Mind the gap: glucocorticoids modulate hippocampal glutamate tone underlying individual differences in stress susceptibility. Mol Psychiatry 20:755–763. 10.1038/mp.2014.96 25178162 PMC4366364

[B60] Ono M, Kikusui T, Sasaki N, Ichikawa M, Mori Y, Murakami-Murofushi K (2008) Early weaning induces anxiety and precocious myelination in the anterior part of the basolateral amygdala of male Balb/c mice. Neuroscience 156:1103–1110. 10.1016/j.neuroscience.2008.07.078 18790016

[B61] Perge JA, Niven JE, Mugnaini E, Balasubramanian V, Sterling P (2012) Why do axons differ in caliber? J Neurosci 32:626–638. 10.1523/JNEUROSCI.4254-11.2012 22238098 PMC3571697

[B62] Phipson B, Lee S, Majewski IJ, Alexander WS, Smyth GK (2016) Robust hyperparameter estimation protects against hypervariable genes and improves power to detect differential expression. Ann Appl Stat 10:946–963. 10.1214/16-AOAS920 28367255 PMC5373812

[B63] Plaisier SB, Taschereau R, Wong JA, Graeber TG (2010) Rank-rank hypergeometric overlap: identification of statistically significant overlap between gene-expression signatures. Nucleic Acids Res 38:e169. 10.1093/nar/gkq636 20660011 PMC2943622

[B64] Purger D, Gibson EM, Monje M (2016) Myelin plasticity in the central nervous system. Neuropharmacology 110:563–573. 10.1016/j.neuropharm.2015.08.001 26282119

[B65] Razzoli M, Carboni L, Andreoli M, Ballottari A, Arban R (2011a) Different susceptibility to social defeat stress of BalbC and C57BL6/J mice. Behav Brain Res 216:100–108. 10.1016/j.bbr.2010.07.014 20654656

[B66] Razzoli M, Carboni L, Andreoli M, Michielin F, Ballottari A, Arban R (2011b) Strain-specific outcomes of repeated social defeat and chronic fluoxetine treatment in the mouse. Pharmacol Biochem Behav 97:566–576. 10.1016/j.pbb.2010.09.010 20863846

[B67] Ritchie ME, Phipson B, Wu D, Hu Y, Law CW, Shi W, Smyth GK (2015) limma powers differential expression analyses for RNA-sequencing and microarray studies. Nucleic Acids Res 43:e47. 10.1093/nar/gkv007 25605792 PMC4402510

[B68] Savignac HM, Finger BC, Pizzo RC, O'Leary OF, Dinan TG, Cryan JF (2011) Increased sensitivity to the effects of chronic social defeat stress in an innately anxious mouse strain. Neuroscience 192:524–536. 10.1016/j.neuroscience.2011.04.054 21635938

[B69] Schulman JJ, Cancro R, Lowe S, Lu F, Walton KD, Llinás RR (2011) Imaging of thalamocortical dysrhythmia in neuropsychiatry. Front Hum Neurosci 5:69. 10.3389/fnhum.2011.00069 21863138 PMC3149146

[B70] Sekiguchi A, Sugiura M, Taki Y, Kotozaki Y, Nouchi R, Takeuchi H, Araki T, Hanawa S, Nakagawa S, Miyauchi CM, Sakuma A, Kawashima R (2014) White matter microstructural changes as vulnerability factors and acquired signs of post-earthquake distress. PLoS One 9:e83967. 10.1371/journal.pone.0083967 24400079 PMC3882214

[B71] Sittig LJ, Carbonetto P, Engel KA, Krauss KS, Barrios-Camacho CM, Palmer AA (2016) Genetic background limits generalizability of genotype-phenotype relationships. Neuron 91:1253–1259. 10.1016/j.neuron.2016.08.013 27618673 PMC5033712

[B72] Smoller JW (2016) The genetics of stress-related disorders: PTSD, depression, and anxiety disorders. Neuropsychopharmacology 41:297–319. 10.1038/npp.2015.266 26321314 PMC4677147

[B73] Subramanian A, Tamayo P, Mootha VK, Mukherjee S, Ebert BL, Gillette MA, Paulovich A, Pomeroy SL, Golub TR, Lander ES, Mesirov JP (2005) Gene set enrichment analysis: a knowledge-based approach for interpreting genome-wide expression profiles. Proc Natl Acad Sci USA 102:15545–15550. 10.1073/pnas.0506580102 16199517 PMC1239896

[B74] Tafti M, Petit B, Chollet D, Neidhart E, de Bilbao F, Kiss JZ, Wood PA, Franken P (2003) Deficiency in short-chain fatty acid beta-oxidation affects theta oscillations during sleep. Nat Genet 34:320–325. 10.1038/ng1174 12796782

[B75] Tanti A, Kim JJ, Wakid M, Davoli MA, Turecki G, Mechawar N (2017) Child abuse associates with an imbalance of oligodendrocyte-lineage cells in ventromedial prefrontal white matter. Mol Psychiatry Advance online publication. Retrieved November 21, 2017. doi:10.1038/mp.2017.231. 10.1038/mp.2017.231 29158585

[B76] Taub AH, Perets R, Kahana E, Paz R (2018) Oscillations synchronize amygdala-to-prefrontal primate circuits during aversive learning. Neuron 97:291–298.e3. 10.1016/j.neuron.2017.11.042 29290553

[B77] Threadgill DW, Dlugosz AA, Hansen LA, Tennenbaum T, Lichti U, Yee D, LaMantia C, Mourton T, Herrup K, Harris RC, et al. (1995) Targeted disruption of mouse EGF receptor: effect of genetic background on mutant phenotype. Science 269:230–234. 7618084 10.1126/science.7618084

[B78] Tovote P, Fadok JP, Lüthi A (2015) Neuronal circuits for fear and anxiety. Nat Rev Neurosci 16:317–331. 10.1038/nrn3945 25991441

[B79] Wang L, Leonards CO, Sterzer P, Ebinger M (2014) White matter lesions and depression: a systematic review and meta-analysis. J Psychiatr Res 56:56–64. 10.1016/j.jpsychires.2014.05.005 24948437

[B80] Vialou V, Bagot RC, Cahill ME, Ferguson D, Robison AJ, Dietz DM, Fallon B, Mazei-Robison M, Ku SM, Harrigan E, Winstanley CA, Joshi T, Feng J, Berton O, Nestler EJ (2014) Prefrontal cortical circuit for depression- and anxiety-related behaviors mediated by cholecystokinin: role of ΔFosB. J Neurosci 34:3878–3887. 10.1523/JNEUROSCI.1787-13.2014 24623766 PMC3951691

[B81] Wittchen HU, Jacobi F, Rehm J, Gustavsson A, Svensson M, Jönsson B, Olesen J, Allgulander C, Alonso J, Faravelli C, Fratiglioni L, Jennum P, Lieb R, Maercker A, van Os J, Preisig M, Salvador-Carulla L, Simon R, Steinhausen HC (2011) The size and burden of mental disorders and other disorders of the brain in Europe 2010. Eur Neuropsychopharmacol 21:655–679. 10.1016/j.euroneuro.2011.07.018 21896369

[B82] Xiao Y, Hsiao TH, Suresh U, Chen HI, Wu X, Wolf SE, Chen Y (2014) A novel significance score for gene selection and ranking. Bioinformatics 30:801–807. 10.1093/bioinformatics/btr671 22321699 PMC3957066

[B83] Young MD, Wakefield MJ, Smyth GK, Oshlack A (2010) Gene ontology analysis for RNA-seq: accounting for selection bias. Genome Biol 11:R14. 10.1186/gb-2010-11-2-r14 20132535 PMC2872874

[B84] Zhang H, Yan G, Xu H, Fang Z, Zhang J, Zhang J, Wu R, Kong J, Huang Q (2016) The recovery trajectory of adolescent social defeat stress-induced behavioral (1)H-MRS metabolites and myelin changes in Balb/c mice. Sci Rep 6:27906. 10.1038/srep27906 PMC4901266 27283029

[B85] Zhang Y, Chen K, Sloan SA, Bennett ML, Scholze AR, O'Keeffe S, Phatnani HP, Guarnieri P, Caneda C, Ruderisch N, Deng S, Liddelow SA, Zhang C, Daneman R, Maniatis T, Barres BA, Wu JQ (2014) An RNA-sequencing transcriptome and splicing database of glia, neurons, and vascular cells of the cerebral cortex. J Neurosci 34:11929–11947. 10.1523/JNEUROSCI.1860-14.2014 25186741 PMC4152602

