# Supplementary figures and images for: Genetic Control of Myelin Plasticity after Chronic Psychosocial Stress
Source: eNeuro. 2018 Jul 11;5(4):ENEURO.0166-18.2018. doi: 10.1523/ENEURO.0166-18.2018 (PMC6071195; doi:10.1523/ENEURO.0166-18.2018)

**A**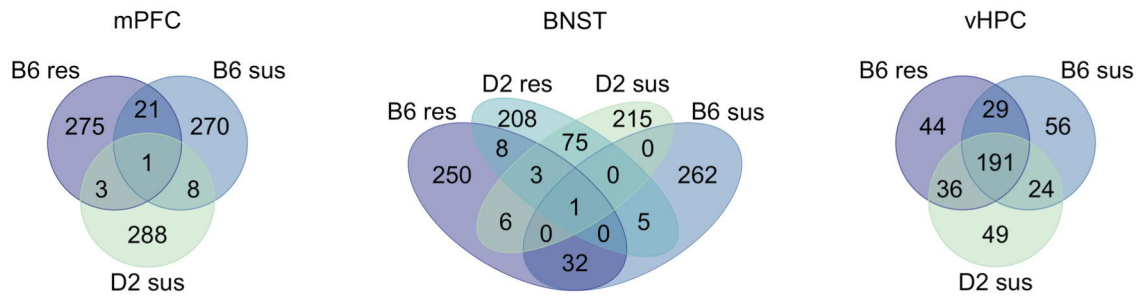**B**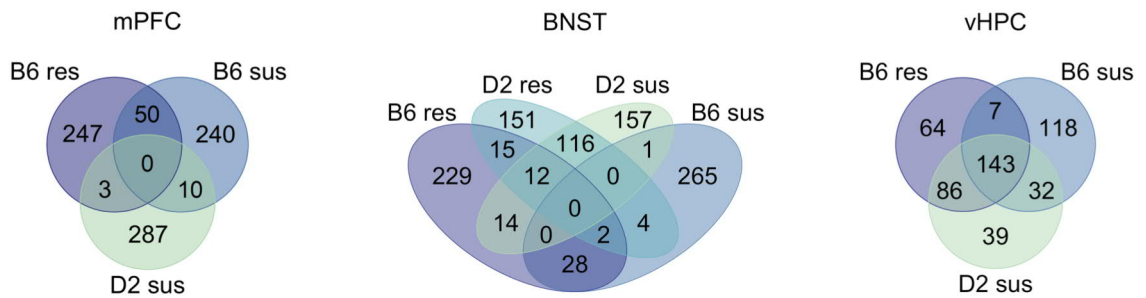**C**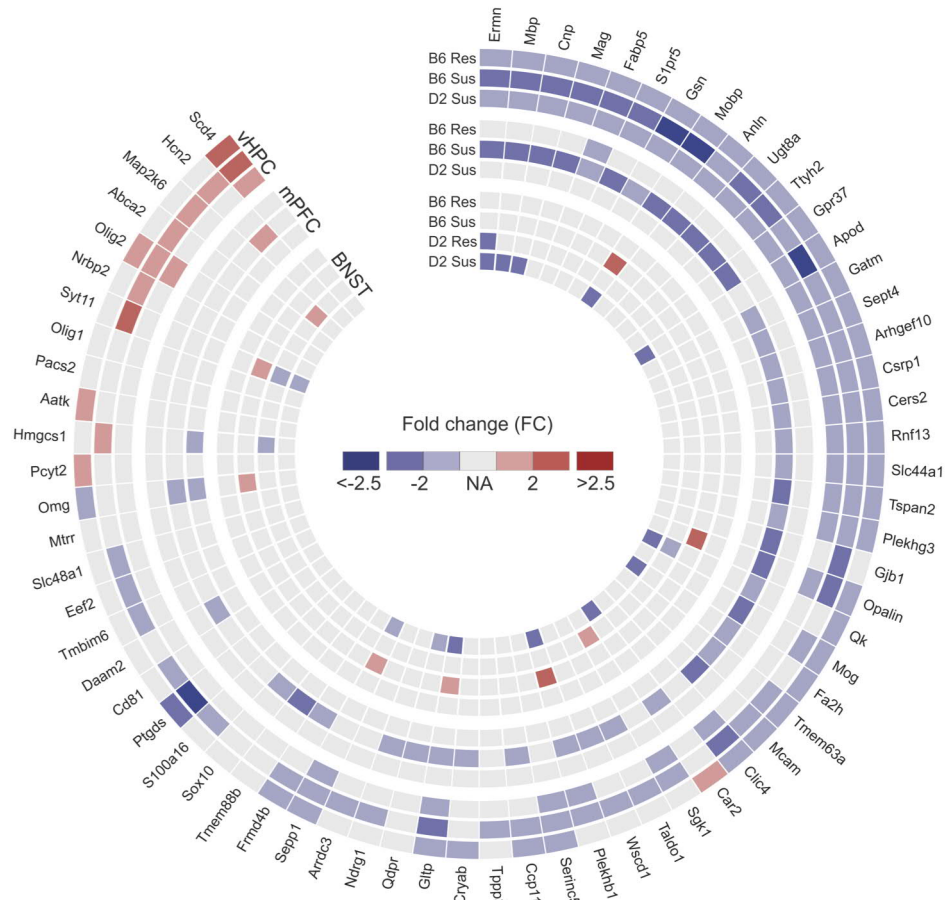

Supplement: Extended Data Figure 2-3 — Overlap of the top differentially expressed and OLG-related genes after CSDS. A, B, Overlap of the top 300 downregulated (A) and top 300 upregulated (B) genes between resilient versus control and susceptible versus control mice, separately for each brain region (Fig. 2B). C, Merged heat map showing the expression fold change (FC) of resilient versus control and susceptible versus control groups. Genes belonging to the LEIN_OLIGODENDROCYTE_MARKERS gene set in the GSEA (Fig. 2E) are shown. FCs are shown only for genes with nominal p < 0.05. Genes which did not pass the cut-off are marked in grey (NA). mPFC: medial prefrontal cortex; vHPC: ventral hippocampus; BNST: bed nucleus of the stria terminalis; B6: C57BL/6NCrl; D2: DBA/2NCrl; Con: control; Res: resilient; Sus: susceptible. Download Figure 2-3, PDF file. [file sup_enu-eN-NWR-0166-18-s06.pdf]

## TEM samples (B6 & D2)

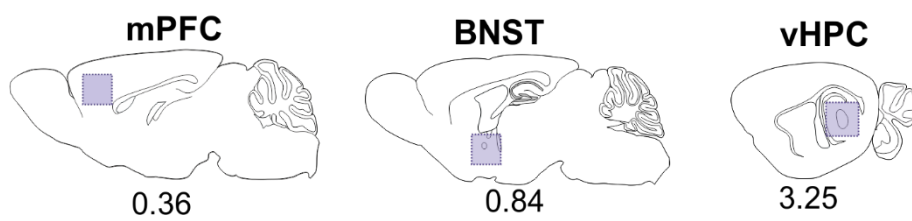

Supplement: Extended Data Figure 5-1 — Regions dissected for TEM. Purple shaded squares outline the dissected samples from 200-µm sections. Atlas outlines are based on Franklin and Paxinos (2008), and the distance from the midline (sagittal) in millimeters is shown below each image. Download Figure 5-1, PDF file. [file sup_enu-eN-NWR-0166-18-s09.pdf]

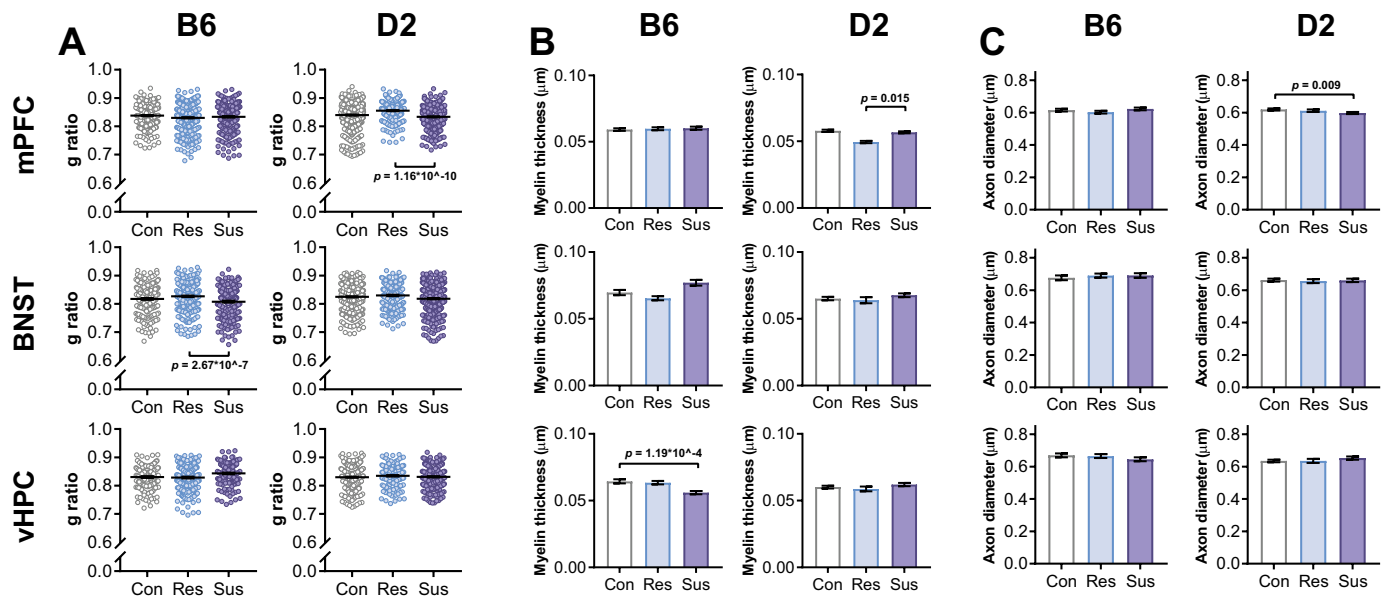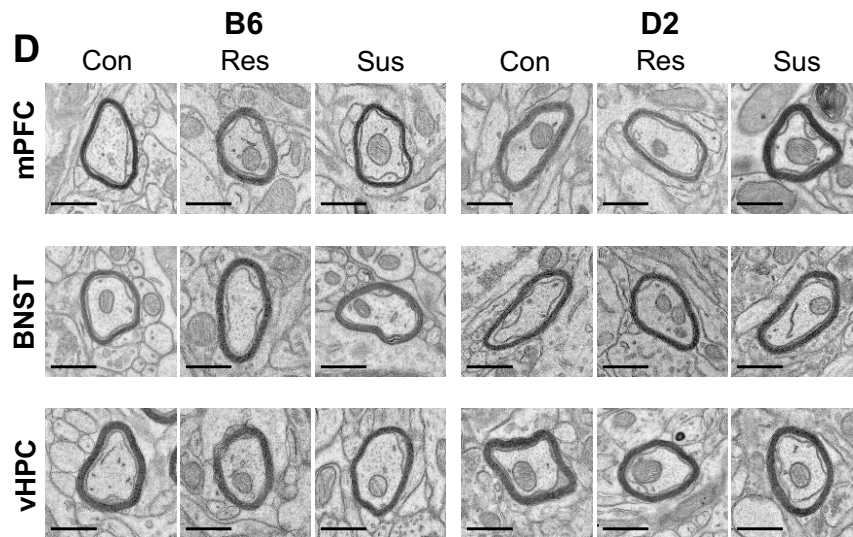

Supplement: Extended Data Figure 5-2 — Effect of CSDS on g ratio (A) and myelin thickness (B) when comparing all axons without division into axon size categories (for results with division into small, medium, and large axon size categories, see Fig. 5) and on axon diameter (C). D, Representative TEM images for each group, scale bar = 0.5 µm. Error bars ± 1 SEM. All nominal p values surviving Bonferroni correction against α = 0.0167 are shown. Download Figure 5-2, PDF file. [file sup_enu-eN-NWR-0166-18-s10.pdf]
